# Benign synthesis of terpene-based 1,4-*p*-menthane diamine

**DOI:** 10.1038/s41598-024-58615-5

**Published:** 2024-04-05

**Authors:** Jonas O. Wenzel, Luis Santos Correa, Sarah Schmidt, Michael A. R. Meier

**Affiliations:** 1 Laboratory of Applied Chemistry, Institute of Organic Chemistry (IOC), Karlsruhe Institute of Technology (KIT), Kaiserstraße 12, 76131 Karlsruhe, Germany; 2 Laboratory of Applied Chemistry, Institute of Biological and Chemical Systems-Functional Molecular Systems (IBCS-FMS), Karlsruhe Institute of Technology (KIT), Kaiserstraße 12, 76131 Karlsruhe, Germany

**Keywords:** Green chemistry, Organic chemistry, Photochemistry

## Abstract

Terpenes represent a promising renewable feedstock for the substitution of fossil resources in the synthesis of renewable platform chemicals, like diamines. This work describes the synthesis and full characterization of 1,4-*p*-menthane diamine (**1,4-PMD**) obtained from *α*-terpinene (**1**). A two-step procedure using dibenzyl azodicarboxylate (DBAD) and H_2_ as rather benign reagents was employed under comparatively mild conditions. Both C–N bonds were formed simultaneously during a visible-light mediated *Diels–Alder* reaction, which was investigated in batch or flow, avoiding regioselectivity issues during the amination steps that are otherwise typical for terpene chemistry. Heterogeneously catalyzed quadruple hydrogenation of the cycloadduct (**2a**) yielded **1,4‑PMD** (**3**). While the intermediate cycloadduct was shown to be distillable, the target diamine can be sublimed, offering sustainable purification methods.

## Introduction

**Figure 1:**
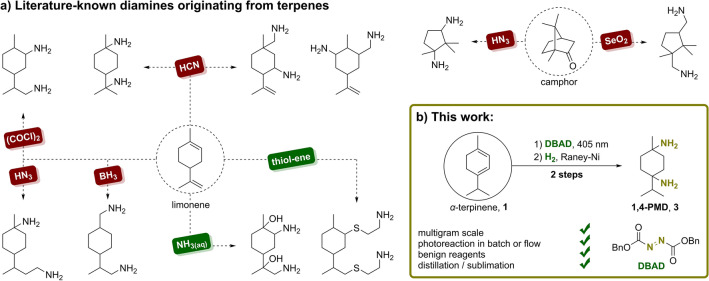
(**a**) Literature known terpene-derived diamines and key reagents used for their syntheses (red/green) are depicted (details in Sect. 2 of the supporting information)^20–35^. (**b**) Synthesis of 1,4-*p*-menthane diamine (**1,4-PMD**, **3**) from *α*-terpinene (**1**) reported in this work.

Primary diamines are organic compounds bearing two primary amino (NH_2_) moieties and represent an essential substance class in pharmacy^1–3^, coordination chemistry, or material sciences^4–7^. Most dominantly, diamines are utilized as monomers of polymeric materials like polyamides (PAs), polyurethanes (PUs) or epoxy resins. Well established representatives are 1,6-diaminohexane, 4,4′-bis(aminophenyl)methylene, or 2,4-diaminotoluene^8–10^. The European market demand of PAs and PUs was around 5 million t/a in 2021, corresponding to ~ 10% of the total European demand for polymeric materials^11^. Most of the industrially produced diamines are based on fossil resources. Commonly, nitrogen-containing moieties are introduced into unsaturated hydrocarbons through oxidation and amination, hydrocyanation or nitration. Following interconversions like reduction or hydrolysis give access to diamines^6,12^. Considering aspects of social, environmental, and economical sustainability, the substitution of fossil resources by renewables should be facilitated, especially for compounds with high production volumes like diamines. Thus, efforts to synthesize diamines from renewable resources like sugars, vegetable oils or amino acids have been described^7,13–15^. Terpenes and terpenoids are discussed as one of the most promising feedstocks for renewable-based polymeric materials^16,17^. However, it should be noted that terpene derived monomers are, with a few exceptions, mainly of interest for specialty applications, for instance if chirality is a desired property. The synthesis of renewable diamines from terpenes is therefore highly desired and was denoted as “worthwhile goal” for organic and industrial chemists^18^. Many procedures were explored to introduce NH_2_ groups into terpenoids, but efficient difunctionalization is often hindered by hard-to-control regio- and stereoselectivity^19^. Hence, only a few diamines based on terpenes are reported in the literature (Fig. 1a)^20–35^. These diamines display valuable surrogates for fossil-based counterparts and found application in polymer and coordination chemistry^16^. Unfortunately, their syntheses were mostly dominated by extremely harsh and dangerous reaction conditions. The well-known 1,8-*p*-menthane diamine for example is synthesized by Ritter reaction using hydrogen cyanide in strong acidic medium^20^, or with acetonitrile in strong acid but with lower yield^27^. Also for two other limonene-derived diamines hydrogen cyanide was the reagent of choice^26^, which is synthetically efficient but connected to severe safety concerns due to toxicity and volatility of HCN. Examples by Rieger or Aldrich-Wright used sodium azide as nitrogen source, which is likewise accompanied by safety concerns due to the explosive character of azides and toxicity of HN_3_ when acidic media are applied^21,34^. It seems clear that circumventing selectivity issues in terpene chemistry is often achieved by using strongly polarized functional groups and reagents to enable only one possible reaction pathway. The drawback hereby often is severe toxicity of the used reactants. Our group reported thiol-ene functionalization of limonene to a diamine in 2013^25^. Furthermore, Mühlhaupt et al*.* reported the synthesis of a limonene-based diamine by epoxide ring opening with aqueous ammonia in 2016^24^. These compounds are valuable terpene-diamines synthesized applying comparatively mild conditions (Fig. 1a). However, they are not solely aliphatic as they further bear hydroxy or thioether moieties, which intrinsically influence the properties of corresponding materials or compounds. Conclusively, the synthesis of purely aliphatic terpene-based diamines in a regio- and stereospecific manner using milder and more benign reaction conditions remains highly desirable. We questioned ourselves if issues of selectivity and efficiency during the amination, typically observed in terpene chemistry, can be avoided if both nitrogen functionalities are introduced in one step. A synthetic tool to achieve concerted double C–N bond formation are di-aza-Diels–Alder reactions of azo compounds with 1,3-dienes, which is a common structural motif in terpenes^26,36–38^.

The obtained cycloadducts can be deprotected at the nitrogen atoms and reduced to cleave the N–N-single bond to yield 1,4-diamines^36^. A few reports spread over the twentieth century hinted at azodicarboxylates (ADCs) as suitable azo derivatives to realize this strategy for the synthesis of diamines^39^. In 2002, Micouin and Bonin described a derivative of 1,3‑diaminocyclopentane synthesized by Diels–Alder reaction and hydrogenation with in-situ benzoylation^40^. To the best of our knowledge, reaction sequences of this overlooked strategy were not investigated and optimized in connection with the synthesis of diamines from renewable feedstocks. ADCs show no distinct hazards like toxicity or explosiveness, if the substituents are large (> C_3_)^41–44^. Additionally, several procedures are described to synthesize ADCs from starting materials like ammonia and carbonates or urea and alcohols by modern reaction methods like electrolysis^45–50^. Although the dominating production process still starts from hydrazine^51^, low hazards and the in principle feasible synthesis from ammonia make ADCs promising and more sustainable surrogates for amination reagents like HCN or HN_3_^52^. This work thus reports on the first synthesis of 1,4-*p*-menthane diamine (**1,4-PMD**, **3**) from renewable *α*-terpinene (**1**) (Fig. 1b), which is obtained by distillation of pine oil or by thermolysis of *α*-pinene^53,54^.

## Results and discussion

**Figure 2:**
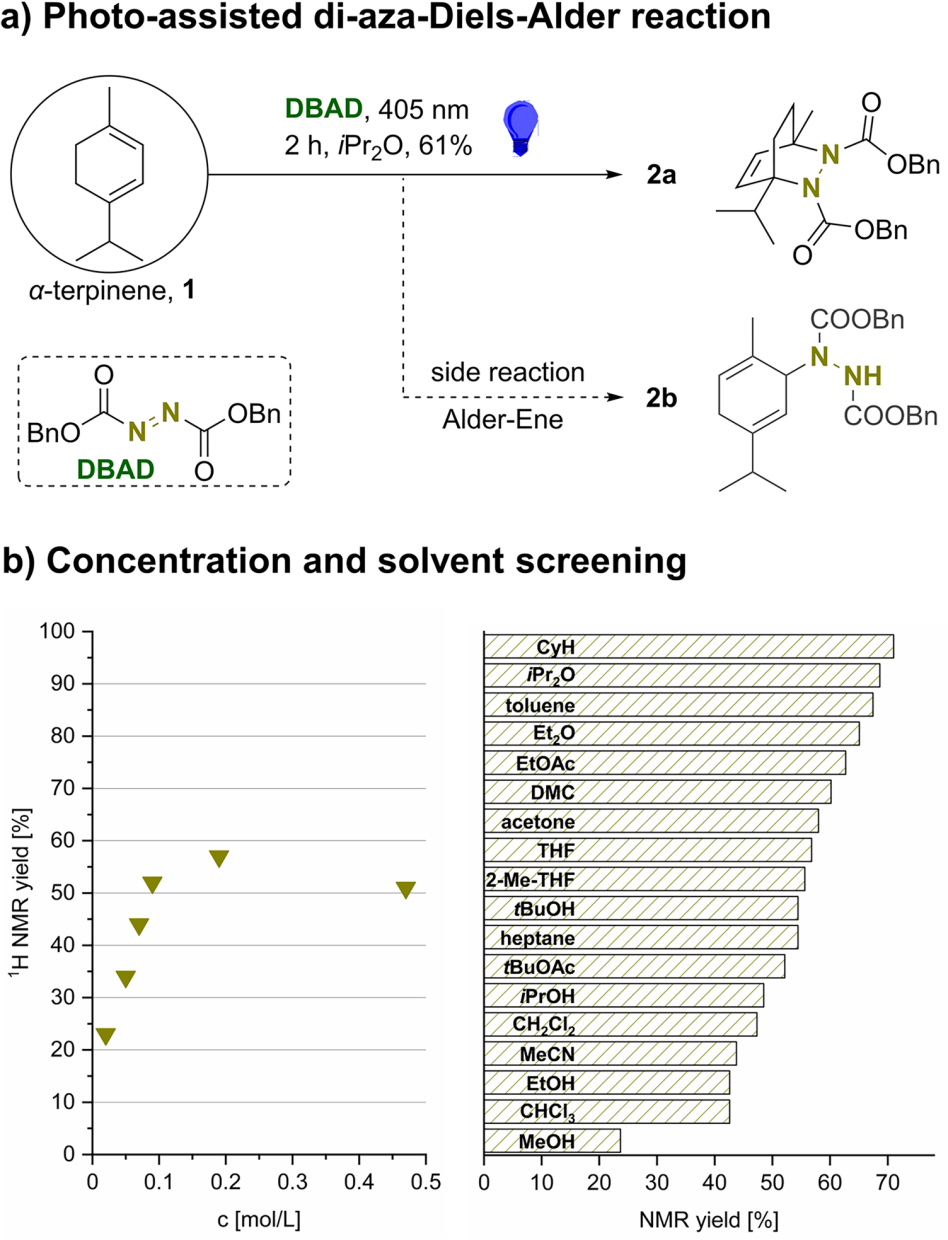
(**a**) Photo-assisted di-aza-Diels–Alder reaction of **1** with DBAD (1.00 equiv. **1**, 1.00 equiv. DBAD, 0.19 mol/L, *i*Pr_2_O, 405 nm, 2 h, 25–38 °C, 61% isolated yield). (**b**) Observed ^1^H NMR yields of the cycloaddition during the optimization process. Left: optimization of concentration (1.00 equiv. **1**, 1.02 equiv. DBAD, acetone, 405 nm, 2 h); right: solvent screening (1.00 equiv. **1**, 1.02 equiv. DBAD, 0.19 mol/L, 405 nm, 2 h).

This work targeted the cycloaddition of *α*-terpinene (**1**) with dibenzyl azodicarboxylate (DBAD) and the subsequent hydrogenation of the corresponding adduct **2a** to yield 1,4-*p*-menthane diamine (**1,4-PMD**, **3**) as exemplary renewable terpene-derived diamine and to demonstrate the general feasibility of this new synthesis route to diamines. We started our investigations with the treatment of **1** with 1.02 eq. of DBAD in the absence of solvent under ambient temperature. This reaction showed full conversion of **1** within one hour, but yielded the cycloaddition product **2a** in only 7% ^1^H NMR yield. Most of the substrate was converted into the Alder-Ene adduct **2b** (Fig. 2a). Conducting the reaction in solution also mainly led to Alder–Ene reaction, but with slower conversion of **1**. Paralleling previous findings from Askani et al., the reaction was repeated under photochemical conditions to accelerate a possible Diels–Alder reaction by photo-isomerizing the azo compound^55^. The absorption maximum of DBAD was determined to 400 nm, which is in good agreement with reports for diisopropyl azodicarboxylate indicating the azo moiety to be the main chromophore^56^. A solution of **1** and DBAD in Et_2_O (0.5 mol/L) was thus irradiated with a 405 nm LED for 92 h within a small crimp vial, while vigorously stirring. The desired Diels–Alder product **2a** was isolated in 38% after chromatographic work-up as colorless oil, while the Alder–Ene adduct **2b** was isolated in 40% yield as colorless solid. The cycloadduct is gathered as mixture of two stereoisomers in 76:24 ratio, which could not be separated. It was suggested that the high activation barrier of C(O)–N rotation leads to the observation of these isomers, as similar phenomena in NMR measurements were already reported for such compounds^57^.

As still significant amounts of **1** were converted into the Alder-Ene product **2b**, we searched for reaction conditions that promote the cycloaddition. We assumed that the used solvent, the applied concentration, and the efficiency of the irradiation are key aspects for achieving higher yields of the Diels–Alder reaction. Reacting **1** with slight excess of DBAD (1.02 eq.) in the absence of light revealed that the Alder–Ene reaction consumes 78% of **1** within 6 h if the reaction was conducted in acetone (0.5 mol/L). Conducting the reaction applying the same concentration of 0.5 mol/L in acetone, but irradiating the reaction vessel with 405 nm for 12 h, gave the Diels–Alder adduct **2a** in 51% NMR yield. A further screening of concentration during the di-aza-Diels–Alder reaction of **1** with DBAD showed optimal conditions at 0.19 mol/L (Fig. 2b, left). To determine the actual required reaction time, the ^1^H NMR yield was followed over time showing full conversion of **1** after 2 h, giving **2a** in 57% NMR yield.

Temperature control revealed that the reaction mixtures warmed up to a constant temperature of 38 °C during irradiation. Cooling the irradiation setup to 20 °C did not show any effect on the reaction yield. Afterwards, the cycloaddition was conducted applying the determined optimal concentration and using 20 different solvents, from which cyclohexane and *i*Pr_2_O showed the highest performance with 69–71% ^1^H NMR yield, respectively (Fig. 2b, right). The reaction tolerates a variety of solvents, while the best ones showed ^1^H NMR yields between 60 and 71%. Solvents giving yields < 60% showed poor solubility of DBAD (e.g. heptane), too high polarity (e.g. MeCN, DMSO) or acidic protons. It is suggested that the latter two types might initiate side reactions with DBAD^58^. With these optimal reaction conditions in hand, **2a** was synthesized from **1** and DBAD in *i*Pr_2_O (0.19 mol/L) by irradiation with 405 nm at 38 °C for 2 h in 61% isolated yield after purification. Fortunately, some of the similarly well-working solvents are quite satisfying by means of sustainable reaction design. Cyclohexane can in theory be synthesized from terpene-derived 1,4-cyclohexadiene, but is not suggested as renewable-based^59,60^. However, cyclohexane has high concerns regarding its flammability and vapor pressure and is therefore undesirable^61^. Although a high yield was achieved in diisopropyl ether, this solvent suffers from the risk of peroxide formation. Dimethyl carbonate, diethyl ether and ethyl acetate are potentially produced from renewable feedstocks like bioethanol or urea^62^. However, diethyl ether shows some serious safety issues due to high volatility, wide explosion ranges and low ignition temperatures^61^. As most promising solvent for the di-aza-Diels–Alder reaction of **1** with DBAD in terms of sustainable reaction design, EtOAc and DMC are suggested as they show a comparably high boiling point, low toxicity, and have less safety concerns. Furthermore, it was shown that it was possible to distill **2a** within a Kugelrohr apparatus at ~ 10^–1^ mbar and 150–170 °C, thus not requiring additional solvents for purification.

**Figure 3:**
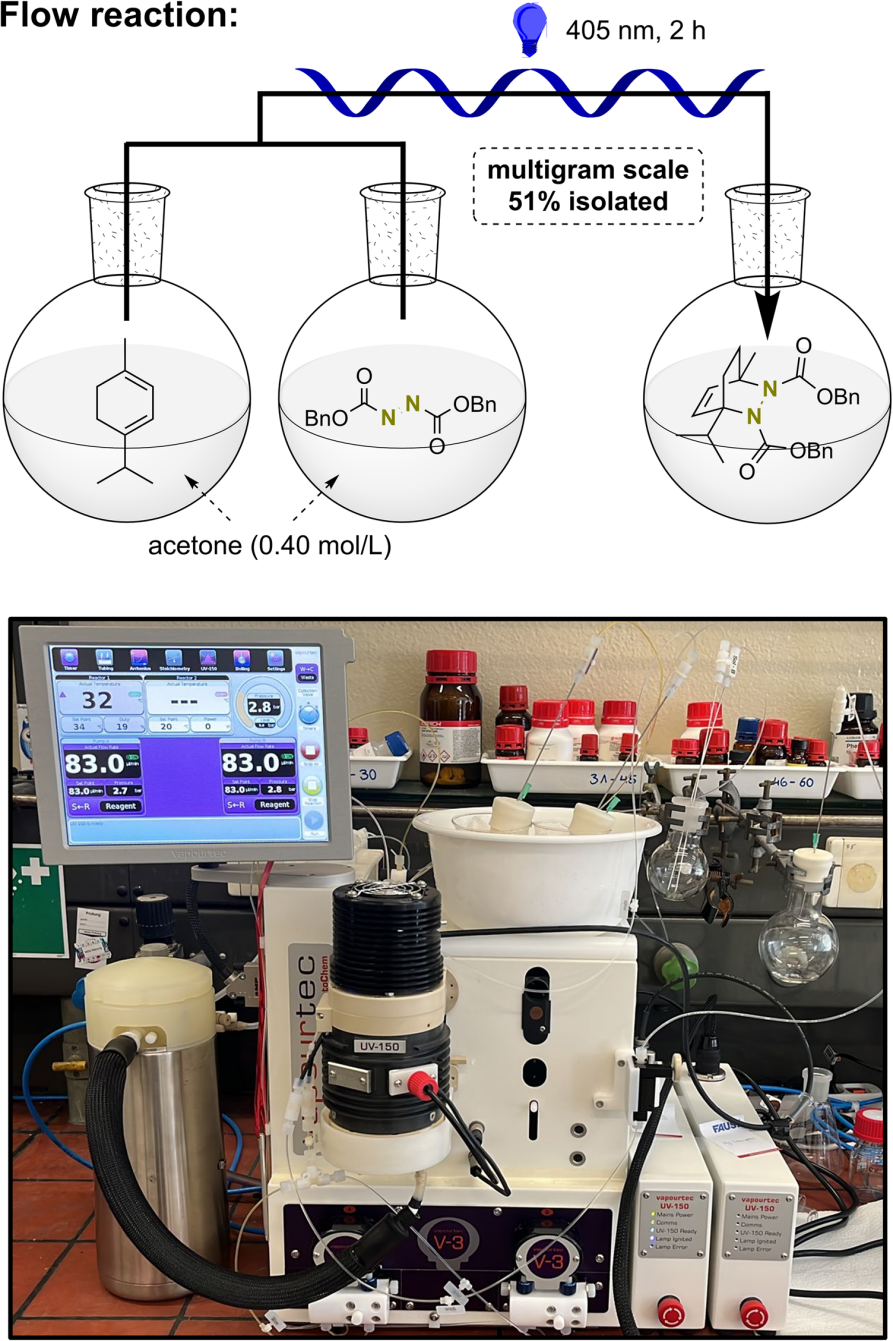
Photo-assisted di-aza-Diels–Alder reaction of **1** with DBAD conducted in a flow reactor enabled isolation of the cycloadduct **2a** in multigram (up to 12.3 mmol) scale (1.00 equiv. **1**, 1.00 equiv. DBAD, 0.20 mol/L, acetone, 405 nm, 2 h, 25–35 °C, 51% isolated yield). Picture taken by author LSC.

Conducting photochemical reactions in batch reactors often limits the potential for upscaling, as efficient irradiation of the reaction mixture requires small amounts of used solvents and rather low concentrations. During the present cycloaddition, the scale was limited to < 600 μmol per batch due to our laboratory setup (see Fig. S1 of Supporting Information). We were thus interested if this limitation can be conquered by conducting the reaction in flow. In flow reactors, the reactants are subjected to the light source within a very thin capillary, thus guaranteeing powerful irradiation. Therefore, two solutions of **1** and DBAD in acetone (each concentration 0.40 mol/L) were reacted by mixing them in a flow reactor and applying a total irradiation time of 2 h, which afforded **2a** in 51% isolated yield on multigram scale (Fig. 3). Acetone was used during the flow reaction instead of *i*Pr_2_O, EtOAc or DMC to avoid partial crystallization/precipitation of DBAD within the reactor as the saturation limit of DBAD in these solvents is ~ 0.20 mol/L.

**Figure 4:**
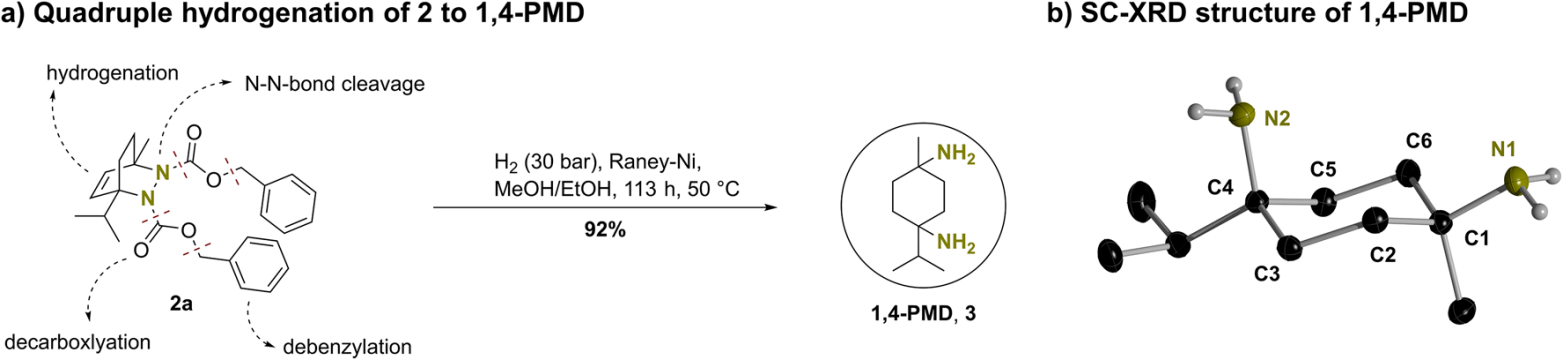
(**a**) Hydrogenation of **2a** to 1,4-*p*-menthane diamine **3** catalyzed by Raney–Nickel (30 bar H_2_, 40 wt% Raney–Ni, MeOH/EtOH (1:1), 113 h, 50 °C, 92% isolated yield). (**b**) SC-XRD structure of **1,4-PMD** (**3**). Carbon-bonded hydrogen atoms and water molecules are omitted for clarity. Thermal ellipsoids are given at the 30% probability level for carbon and nitrogen atoms. Nitrogen-bonded hydrogen atoms are depicted as isotropic spheres. Selected bond lengths in [Å]: N1-C1 1.4772(11), C4-N2 1.4753(11), C1-C2 1.5306(12), C2-C3 1.5283(12), C3-C4 1.5367(12), C4-C5 1.5310(12), C5-C6 1.5236(12), C6-C1 1.5243(12).

After we examined the synthesis of **2a**, the quadruple hydrogenation of the title cycloadduct was targeted to get first access to 1,4-*p*-menthane diamine (**1,4-PMD**). We assumed the benzyl moieties originating from the used azo dicarboxylate to be cleavable by heterogeneously catalyzed hydrogenolysis. The formed bis carbamic acid was expected to decarboxylate twice to give the bicyclic hydrazine derivative of the terpinene scaffold. Such cyclic hydrazines were reported to undergo N–N single bond cleavage using heterogeneous hydrogenation conditions^39,40^. The C = C double bond of the cycloadducts backbone was also expected to be reduced by heterogeneously catalyzed hydrogenation (Fig. 4a).

Therefore, we challenged this synthetic strategy of quadruple hydrogenation by treating **2a** with 100 wt% of Raney–Nickel (25 wt% per to be hydrogenated functional group) in a 1:1 mixture of MeOH/EtOH and 30 bar of H_2_. This mixture was stirred for 134 h at 60 °C. Afterwards, the catalyst was filtered off and the solvent was removed, affording a colorless crystalline solid, which was well soluble in dichloromethane and partly soluble in water. This crude product was confirmed to consist of the desired diamine by NMR and IR spectroscopy as well as ASAP mass spectrometry. The mass of the obtained product corresponded to quantitative yield of **1,4-PMD**. According to ^1^H NMR spectroscopy, the crude product already showed high purity, but ~ 32 mol% of **3** were present as the ammonium formate salt ([**3**H][HCOO]) and ~ 16 mol% as the ammonium acetate salt ([**3**H][CH_3_COO]). It is expected that formate is probably formed by CO_2_ reduction catalyzed by Raney–Nickel^63^ and acetate is formed by hydrogenolysis of trace impurities of EtOAc in the starting material. **3** was purified by dissolving it in diluted hydrochloric acid and washing of the aqueous phase with dichloromethane. Bringing the aqueous phase to pH > 12 and extracting it with dichloromethane afforded the diamine in high purity without the presence of formate or acetate salts. After this aqueous workup, suitable single crystals of **1,4-PMD** were obtained from a supersaturated CH_2_Cl_2_ solution (see Sect. 5, Supporting Information). The compound crystallizes with two equivalents of water in the solid structure (**3**·2 H_2_O) in the monoclinic space group C2/c (Fig. 4b). All C–C bonds within the six-membered ring have almost the same length (1.52–1.53 Å), underlining full saturation of the former unsaturated scaffold. Obviously, only the cis-isomer of **3** is formed due to the stereospecific nature of the initial cycloaddition. In crystalline solid state, **1,4-PMD** does exclusively adopt one of two possible conformers of the cyclohexane scaffold, in which the isopropyl moiety at C4 acts as anchoring group claiming equatorial position. Disordered hydrogen bonds between the amine functionalities and the incorporated water molecules were observed and resulted furthermore in the disappearance of NH signals in ^1^H NMR spectra if the compound was purified by aqueous work-up even after intense drying. Therefore, the exact content of water could not be determined. Assuming a remaining water content of 0–2 equivalents, the isolated yield of **3** corresponded to 82–91%. After this initial isolation of **1,4-PMD**, the catalyst loading was lowered to 40 wt% (10 wt% per hydrogenated functional group) giving **3** in 92% isolated yield after 113 h reaction time at 50 °C and after purification. This procedure was applicable in multigram scale, in which ~ 5 g of **2a** were successfully converted into **1,4-PMD**. Furthermore, it was observed that the diamine **3** starts to sublime at 80 °C and ambient pressure, which is suggested to enable possible solvent-free purification procedures, thus reducing waste production during purification.

## Conclusion

Terpenes are one of the most promising renewable feedstocks for the synthesis of platform chemicals. This work targeted the synthesis of 1,4-*p*-menthane diamine (**1,4-PMD**) from *α*-terpinene (**1**) in two steps. A thorough screening of reaction conditions enabled the facile synthesis of the intermediate **2a** by di-aza-Diels–Alder reaction assisted by visible light. Further heterogeneously catalyzed quadruple hydrogenation yielded **1,4-PMD** in excellent yield. The obtained diamine was fully characterized by means of NMR and IR spectroscopy, mass spectrometry as well as SC-XRD. Both steps were conducted in multigram scale, facilitated by subjecting the optimized cycloaddition reaction to flow conditions. While the cycloadduct **2a** was shown to be distillable under reduced pressure, the title diamine can be sublimed at ambient pressure at temperatures above ~ 80 °C. The required reaction conditions for the synthesis **1,4-PMD** do not rely on highly toxic reagents like HCN or HN_3_, which dominate the described syntheses of terpene-based diamines so far. Conclusively, 1,4-*p*-menthane diamine adds to the repertoire of renewable aliphatic diamines. As revealed by SC-XRD measurements, the isopropyl moiety acts as anchor for the conformational array of the six-membered ring. We hope that the synthetic access to **1,4-PMD** under benign reaction conditions will initiate a broader investigation of its polymers and coordination compounds and will contribute to the field of renewable based diamines and their potential applications.

## Experimental section

Detailed information about all used compounds, methods and syntheses can be found in the supporting information. If not stated otherwise, all used compounds were purchased from chemical suppliers and used without further purification. The key starting material of this work, *α*-terpinene (**1**), was purchased from Alfa Aesar and the purity was determined by GC and NMR experiments to be 73%. The compound was used without further purification but the used masses and volumes given in the synthetic procedures correspond to the actual included amount of **1**. Hydrogenations under high pressures were conducted in a 300 mL Berghof pressure reactor with Teflon inlet. Via an integrated thermometer the temperature was monitored interactively during the reaction. The pressure reactor was placed on a magnet stirrer, to enable stirring of the reaction mixture. All reactions under UV irradiation were conducted using one of two LED-arrays with the same principal setup, but different sizes. Both setups consist of a magnetic stirrer, an aluminum cooling element with LEDs attached to it and a vial block with six or five holders. The reaction vessels were placed in the holders and stirring bars inside the vessels enabled homogenization of the reaction mixture through the magnetic stirrer. Holes on the bottom of each holder facilitated irradiation by LEDs, which are placed beneath the holders on the cooling element ensuring a LED-vial distance of 1 cm. The LEDs were serially connected to a constant current transformer, applying a current of 700 mA. The used LEDs were Nichia NVSU119C 2.2 W 405 nm-LEDs on 10 × 10 mm plates. Photo reactions in flow were conducted using an E-series flow reactor from Vapourtec with photochem equipment. As irradiation source, a 405 nm LED with 9 W power was used. The inner volume of the reactor capillary is 10 mL, wherefore a pumping speed of 82.4 µL/min was applied to achieve an irradiation time of the reaction mixture of 2 h. Within this work, the term “freshly prepared Raney–Nickel” refers to elementary Nickel, which was obtained by leaching of a nickel/aluminum alloy (1:1 weight proportion) and was not stored longer than 2 weeks (details in supporting information). NMR Spectroscopy was performed using a Bruker Ascend 400 or Bruker Ascend 500 spectrometer, applying constant temperatures of 298 K. FT-IR measurements were conducted, using a Bruker Alpha FTIR spectrometer with Platinum technology. Each measurement consisted of one background measurement and one transmittance measurement with 24 scans. ASAP-MS measurements were conducted using an Advion expression CMS system.

**2a**: Batch (six reactions per batch): Six reaction mixtures were prepared as follows. 20.8 µL of **1** (73% pure, 12.8 mg, 94.0 µmol, 1.00 equiv.) and 29.1 mg DBAD (96% pure, 27.9 mg, 94.0 µmol, 1.00 equiv.) were dissolved in 0.5 mL of *i*Pr_2_O. The mixtures were irradiated while stirring in 5 mL headspace flasks for 2 h. All six reaction mixtures were combined and the solvent was removed under reduced pressure. The residue was purified by flash column chromatography on silica gel using a gradient from 0 to 20% ethyl acetate in cyclohexane. The product was obtained as colorless oil (149 mg, 342 µmol, 61%). Flow: 5.33 mL **1** (73% pure, 3.27 g, 24.0 mmol, 1.00 equiv.) were dissolved in 60.0 mL acetone and 7.146 g DBAD (96% pure, 7.16 g, 24.0 mmol, 1.00 equiv.) were dissolved in 60.0 mL acetone. Both mixtures were mixed within the flow capillary of a flow reactor and irradiated in flow. The inner volume of the reactor capillary was 10 mL, wherefore a pumping speed of 82.4 µL/min was applied with a mixing ratio of 1:1 to achieve an irradiation time of the reaction mixture of 2 h. Afterwards the solvent was removed under reduced pressure and the residue was purified by flash column chromatography on silica gel using a gradient from 0 to 20% ethyl acetate in cyclohexane. The product was obtained as colorless oil (5.33 g, 12.3 mmol, 51%). To show that **2a** can be distilled, a pure sample of **2a** was filled into a Kugelrohr apparatus. When a pressure of ~ 10^–1^ mbar was applied, the compound starts to distill at 150 °C, while reasonable distillation speeds were observed at temperatures up to 170 °C. The product was obtained as mixture of two diastereomers (rotamers of the C–N-axis).

^**1**^**H NMR** (400 MHz, CDCl_3_, ppm) *δ* = 7.25–7.17 (m, 10H, H_arom._^1^), 6.62 (d, *J* = 8.3 Hz, 1H, CH^2^), 6.25 (d, *J* = 8.3 Hz, 1H, CH^3^), 5.24–4.92 (m, 4H, CH_2_^4^), 3.30 (hept, *J* = 6.9 Hz, 1H, CH^5^), 2.27–2.17 (m, 1H, CH_2_^6^), 1.85 (s, 3H, CH_3_^7^), 1.83–1.72 (m, 1H, CH_2_^6^), 1.45–1.22 (m, 2H, CH_2_^6^), 0.94 (d, *J* = 7.2 Hz, 3H, CH_3_^8^), 0.90 (d, *J* = 7.0 Hz, 3H, CH_3_^8^). ^**13**^**C NMR** (101 MHz, CDCl_3_, ppm) *δ* = 159.33 (C=O^9^), 158.78 (C=O^9^), 157.91 (C=O^9^), 157.58 (C=O^9^), 142.32 (C=C^2^), 140.27 (C=C^3^), 138.81 (C=C^2^), 136.99 (C_quart_.^10^), 136.79 (C_quart_.^10^), 136.68 (C_quart_.^10^), 136.29 (C_quart_.^10^), 131.95 (C=C^3^), 128.47 (C_arom._^1^), 128.42 (C_arom._^1^), 127.95 (C_arom._^1^), 127.87 (C_arom._^1^), 127.80 (C_arom._^1^), 127.76 (C_arom._^1^), 127.72 (C_arom._^1^), 127.51 (C_arom._^1^), 127.37 (C_arom._^1^), 127.12 (C_arom._^1^), 68.35 (C_quart_.^11^), 67.50 (CH_2_^4^), 67.14 (CH_2_^4^), 67.04 (CH_2_^4^), 66.14 (C_quart_.^11^), 59.84 (C_quart_.^12^), 58.31 (C_quart_.^12^), 32.04 (CH_2_^6^), 31.03 (CH_2_^6^), 30.21 (CH_2_^6^), 30.04 (CH^5^), 24.12 (CH_3_^7^), 18.49 (CH_3_^8^), 18.13 (CH_3_^8^), 17.89 (CH_3_^8^), 16.70 (CH_3_^8^). **FTIR**: ν (cm^−1^) = 1701.06 (C=O). **FAB-HRMS**: *m/z* (calc.) = 435.2278 [M + 1^**·**+^]; *m/z* (found) = 435.2278 [M + 1^**·**+^]; (C_26_H_31_N_2_O_4_).

**2b**: This compound was isolated once as side product during the synthesis of **2a** to get full characterization data of the title compound. The irradiation described in the following procedure is not necessary for the synthesis of **2b**. Six batches were prepared according to the following procedure: 300 mg of dibenzyl azodicarboxylate (1.00 mmol, 1.00 eq.) were dissolved in 2.00 mL of dry diethyl ether in a 5 mL headspace flask. 163 µL of *α*-terpinene (137 mg, 1.00 mmol, 1.00 eq.) were added and the flask was sealed with a septum cap. The mixture was stirred at 20 °C for 92 h, while irradiated by 405 nm LEDs. All six batches were combined and the solvent was removed under reduced pressure. The remaining residue was purified by flash column chromatography on silica gel using a gradient from 0 to 20% ethyl acetate in cyclohexane. The product was obtained as colorless solid (873 mg, 2.01 mmol, 40%).

^**1**^**H NMR** (400 MHz, DMSO-*d*_*6*_, ppm) *δ* = 9.37–8.66 (m, 1H, NH^1^), 7.46–7.19 (m, 10H, H_arom._^2^), 5.83–5.57 (m, 1H, CH^3^), 5.51–5.23 (m, 1H, CH^4^), 5.17–4.96 (m, 5H, CH^5^, CH_2_^6^), 2.57–2.40 (m, 2H, CH_2_^7^), 2.24–2.04 (m, 1H, CH^8^), 1.83–1.44 (m, 3H, CH_3_^9^), 1.08–0.75 (m, 6H, CH_3_^10^). ^**13**^**C NMR** (101 MHz, DMSO-*d*_*6*_, ppm) *δ* = 156.33 (C=O^11^), 155.73 (C=O^11^), 144.70 (C_quart._^12^), 136.54 (C_quart._^13^_._), 128.29 (C_arom._^2^), 128.26 (C_arom._^2^), 127.99 (C_arom._^2^), 127.88 (C_arom._^2^), 127.69 (C_arom._^2^), 127.60 (C_arom._^2^), 127.38 (C_arom._^2^), 127.11 (C_arom._^2^), 124.72 (CH^3^), 115.44 (CH^4^), 66.74 (CH_2_^6^), 65.75 (CH_2_^6^), 56.20 (CH^5^), 33.50 (CH^8^), 27.28 (CH_2_^7^), 20.83 (CH_3_^10^), 19.64 (CH_3_^9^). **FTIR**: ν (cm^−1^) = 3274.60 (br, NH), 1742.20 (C=O), 1672.27 (C=O). **FAB-HRMS**: *m/z* (calc.) = 435.2278 [M + 1^**·**+^]; *m/z* (found) = 435.2276 [M + 1^**·**+^]; (C_26_H_31_N_2_O_4_).

**3**: 5.24 g of **2a** (12.1 mmol, 1.00 eq.) were dissolved in 20.0 mL MeOH/EtOH (1:1) in a Teflon inlet, which was inserted into a pressure-reactor. 18.0 mL of a freshly prepared Raney-Nickel slurry of concentration 116 mg/mL were added (2.09 g, 40.0 wt%). The mixture was stirred for 113 h at 50 °C applying a H_2_-pressure of 30 bar. The catalyst was filtered off and the solvent was removed under reduced pressure. The residue was suspended in 20.0 mL of water and acidified with 3.00 mL of concentrated hydrochloric acid. The aqueous phase was washed three times with 50.0 mL CH_2_Cl_2_ and treated afterwards with 10.0 mL of 10.0 mol/L NaOH_(aq)_. The aqueous phase was extracted ten times with 100 mL CH_2_Cl_2_. The combined organic phase was dried over sodium sulfate and the solvent was removed under reduced pressure. The product was obtained as colorless solid (1.88 g, 11.0 mmol, 92%).

^**1**^**H NMR** (400 MHz, CDCl_3_, ppm) *δ* = 1.89 (s, br., NH_2_^1^), 1.63–1.35 (m, 9H, CH^2^, CH_2_^3^), 1.05 (s, 3H, CH_3_^4^), 0.87 (d, *J* = 6.9 Hz, 6H, CH_3_^5^). ^**13**^**C NMR** (101 MHz, CDCl_3_, ppm) *δ* = 51.76 (C_quart._^6^), 48.63 (C_quart._^7^), 36.75 (CH_2_^3^), 36.65 (CH^2^), 32.84 (CH_2_^3^), 26.82 (CH_3_^4^), 16.67 (CH_3_^5^). **FTIR**: ν (cm^−1^) = 3276,65 (NH-stretch, br), 1592,05 (NH_2_-bend). **EI-HRMS**: *m/z* (calc.) = 171.1856 [M + 1^**·**+^]; *m/z* (found) = 171.1860 [M + 1^**·**+^]; (C_10_H_23_N_2_). CCDC number: 2325396.

### Supplementary Information

Supplementary Information.
