# Supplementary material for: Benign synthesis of terpene-based 1,4-p-menthane diamine
Source: Sci Rep. 2024 Apr 5;14:8055. doi: 10.1038/s41598-024-58615-5 (PMC10997780; doi:10.1038/s41598-024-58615-5)
Supplement: Supplementary file 1 — Supplementary Information. [file 41598_2024_58615_MOESM1_ESM.pdf]

Supporting Information for:

Benign synthesis of terpene-based 1,4-*p*-menthane diamine

Jonas O. Wenzel,[†,1] Luis Santos Correa,[†,2] Sarah Schmidt,[2] Michael A. R. Meier[1,2,*]

[†] The authors contributed equally to this work.

1) Laboratory of Applied Chemistry, Institute of Organic Chemistry (IOC), Karlsruhe Institute of Technology (KIT), Kaiserstraße 12, 76131 Karlsruhe (Germany). *) E-Mail: m.a.r.meier@kit.edu; Web: www.meier-michael.com
2) Laboratory of Applied Chemistry, Institute of Biological and Chemical Systems – Functional Molecular Systems (IBCS-FMS), Karlsruhe Institute of Technology (KIT), Kaiserstraße 12, 76131 Karlsruhe (Germany).

Content

Content 1
1 General methods 2
  1.1 Used solvents 2
  1.2 Used compounds 2
  1.3 Flash column chromatography 2
  1.4 Reactions applying H_2 pressure 2
  1.5 Reaction under UV irradiation 2
  1.6 Preparation and use of Raney-Ni 4
  1.7 Thin layer chromatography 5
  1.9 NMR spectroscopy 5
  1.10 Fourier transformation infrared spectroscopy (FT-IR) 6
  1.11 High resolution mass spectrometry 6
  1.12 Atmospheric solids analysis probe mass spectrometry (ASAP-MS) 6
  1.13 Single-crystal X-ray diffractometry (SC-XRD) 6
  1.14 UV/VIS measurements of dibenzyl azodicarboxylate 7
2 Literature reports on terpene-derived diamines 8
3 Optimization of the di-aza-Diels-Alder reaction of 1 with DBAD 11
4 Spectra 16
  4.1 NMR spectra 16
  4.2 IR spectra 28
5 Crystallographic data 31
6 References 33

1 General methods

1.1 Used solvents

All used solvents, except for cyclohexane and ethyl acetate, were used in technical grade without further purification. Deuterated solvents were purchased from Eurisotop and used without further purification.

1.2 Used compounds

If not stated otherwise, all used compounds were purchased from chemical suppliers and used without further purification. The key starting material of this work, *α*-terpinene (**1**), was purchased from Alfa Aesar and the purity was determined by GC and NMR experiments to be 73%. The compound was used without further purification, but the used masses and volumes given in the synthetic procedure correspond the actual included amount of **1**.

1.3 Flash column chromatography

The purification of compounds by flash column chromatography proceeded referring to the idea of *W. C. Still et al.*[2] As stationary phase, silica of technical grade with 60 Å pore size, 230–400 mesh size and 40–63 µm particle size from Sigma Aldrich was used. The eluent is given for each compound separately in section 3. As apparatus, glass columns with integrated frits of different pore sizes and valves were used. Applied pressures for faster flows were achieved by manual pumps.

1.4 Reactions applying H_2 pressure

Hydrogenations under high pressures were conducted in a 300 mL Berghof pressure reactor with Teflon inlet. *Via* an integrated thermometer, the temperature was monitored during the reaction. The pressure reactor was placed on a magnet stirrer, to enable stirring of the reaction mixture.

1.5 Reaction under UV irradiation

All reactions under UV irradiation were conducted using one of two LED-arrays with the same principal setup, but different sizes. Both setups consist of a magnetic stirrer, an aluminum cooling element with LEDs attached to it and a vial block with six (**Figure S1**, left) or five (**Figure S1**, right) holders. The reaction vessels were placed in the holders and stirring bars inside the vessels enabled homogenization of the reaction mixture through the magnetic stirrer. Holes on the bottom of each holder facilitated irradiation from beneath by LEDs, which are placed beneath the holders on the cooling element ensuring a LED to vial distance of 1 cm. The LEDs were serially connected to a constant current transformer, applying a current of 700 mA. The used LEDs were Nichia NVSU119C 2.2 W 405 nm-LEDs on 10×10mm circuit boards.

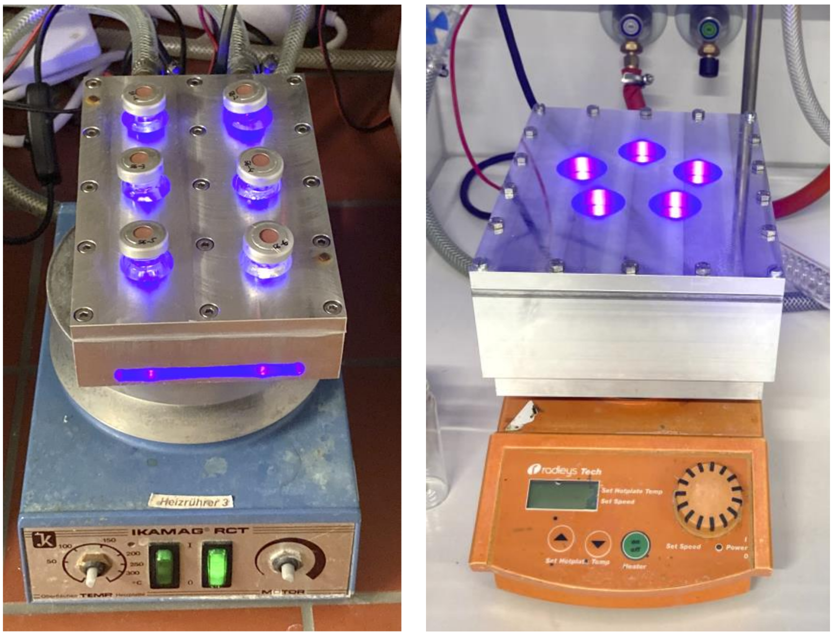

Figure S1. LED-reactors used for reactions under 405 nm irradiation.

Photo reactions in flow were conducted using an E-series flow reactor from Vapourtec with photochem equipment. As irradiation source, a 405 nm LED with 9W power was used. The inner volume of the reactor capillary is 10 mL, wherefor a pumping speed of 82.4 µL/min was applied to achieve an irradiation time of the reaction mixture of 2 hours.

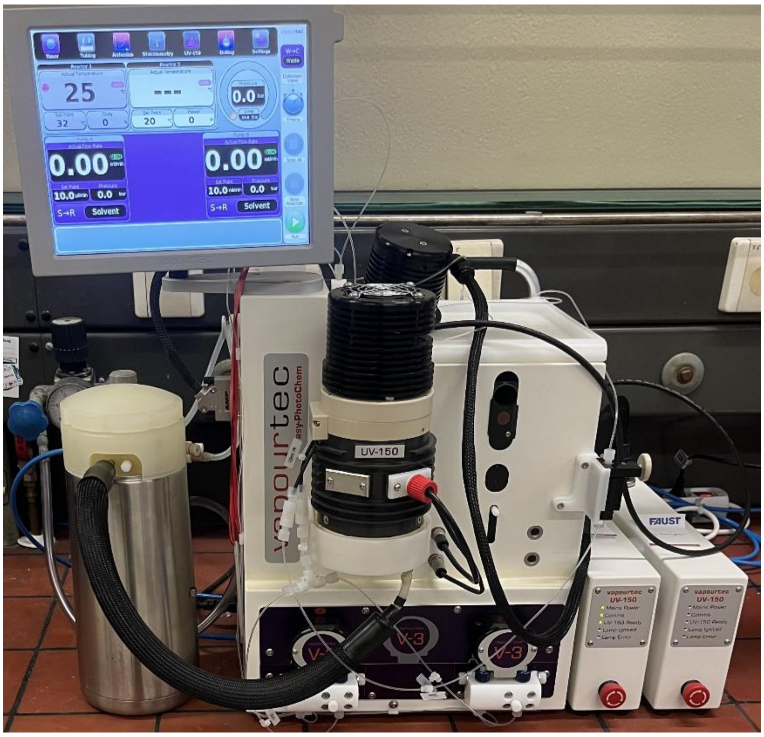

Figure S2. Flow reactor by Vapourtec used in this work.

1.6 Preparation and use of Raney-Ni

Within this work, the term “freshly prepared Raney-Nickel” refers to elementary Nickel, which was obtained by leaching of a nickel/aluminum alloy (1:1 weight proportion) and was not stored longer than for two weeks.

For the preparation of freshly prepared Raney-Nickel, 20.3 g Ni/Al alloy were carefully added portion wise to 220 mL of 3.64 mol/L $NaOH_{(aq)}$ at 80 °C under vigorous stirring. This proceeded slowly, due to strong exothermic gas development (H_2 ↑). After the alloy was added completely to the NaOH solution, the mixture was further stirred at 80 °C until no gas development was observed anymore (~20 minutes) to ensure full oxidation and dissolution of aluminum. The aqueous suspension was cooled to room temperature, decanted, and the resulting solid was washed three times with deionized water and three times with ethanol (HPLC grade) subsequently. After the last washing step, the obtained black nickel was suspended in 87.3 mL ethanol, yielding a nickel/ethanol slurry with a concentration of 0.116 g/mL.

The nickel/ethanol slurry was shaken vigorously before it was added to reaction mixtures, to warrant homogeneity and constant concentration of the slurry. The slurry was stored in a polyethylene screw-cap container, which was sealed additionally with parafilm, to avoid evaporation of the solvent, thus alterations of concentration.

1.7 Thin layer chromatography

TLC measurements were conducted on silica gel coated aluminum plates of type F254, supplied by Sigma Aldrich. After spotting of the compounds on the TLC plates, the plates were placed in a glass chamber filled with a filling level of 0.5 cm of eluent. After one iteration of eluent flow, the compounds spots were visualized by UV light (254 or 365 nm), by $KMnO_4$ solution or by Seebach stain. For determination of the R_f-values of unreported compounds, 1 mg of the isolated substance was dissolved in 1 mL of CH_2Cl_2 and spotted slightly.
The used staining solutions were prepared as follows:

$KMnO_4$-solution: 1.5 g $KMnO_4$, 10 g K_2CO_3, 1.25 mL 10% $NaOH_{(aq)}$, 200 mL H_2O

Seebach stain: 2 g $Ce(SO_4)_2$, 5 g $H_3[P(Mo_3O_{10})_4] \cdot \times H_2O$, 16 mL conc. H_2SO_4, 200 mL H_2O

1.9 NMR spectroscopy

NMR Spectroscopy was performed using a Bruker Ascend 400 or Bruker Ascend 500 spectrometer, applying constant temperatures of 298 K. For sample preparation, 5-20 mg of the compounds were dissolved in deuterated solvents and shaken vigorously to ensure homogenization. If not stated otherwise, ^{1}H-experiments were conducted at 400.13 MHz, with 16 scans and D1=1.00 s. ^{13}C-experiments were conducted at 100.62 MHz, with 1024 scans and D1=2.00s. $^{1}H^{1}H$-correlation-experiments were conducted using gradient selected correlation spectroscopy (gCOSY) or nuclear Overhauser effect spectroscopy (NOESY), $^{1}H^{13}C$-correlation-experiments were conducted using phase-edited heteronuclear single quantum coherence ($HSQC_{ed}$) or heteronuclear multiple bond correlation (HMBC). The chemical shifts (δ) in NMR spectra were given in parts per million (ppm) relative to the chemical shift of tetramethylsilane (TMS), while the chemical shift of TMS was set to 0.00. Furthermore, the chemical shifts were referenced indirectly relative to the solvent, which was used for the measurement, applying the values of *Fulmer et al*.[3] The given signals for each compound were named according to their splitting pattern as follows: s (singlet), d (doublet), t (triplet), q (quartet), p (pentet), sext (sextet), sept (septet), dd (doublet of doublets), ddd (doublet of doublet of doublets), dt (doublet of triplets), m (multiplet), br (broad). Coupling constants (J) were given in Hz.

1.10 Fourier transformation infrared spectroscopy (FT-IR)

FT-IR measurements were conducted using a Bruker Alpha FTIR spectrometer with Platinum technology. Each measurement consisted of one background measurement and one transmission measurement with 24 scans. The IR-spectra show the bands of the investigated compounds as transmittance from 0-1 relative to the wavenumber in cm^{-1} from 4,000 to 400 cm^{-1}, due to convention.

1.11 High resolution mass spectrometry

HRMS measurements were conducted on a Finnigan MAT 95 spectrometer using fast atom bombardment ionization (FAB).

1.12 Atmospheric solids analysis probe mass spectrometry (ASAP-MS)

ASAP-MS measurements were conducted using an Advion expression CMS system.

1.13 Single-crystal X-ray diffractometry (SC-XRD)

Diffraction data were measured using a Stoe IPDS II diffractometer and graphitemonochromated MoKα (0.71073 Å) radiation. Absorption corrections were carried out using the STOE LANA software package.[4] Structure solution were carried out using OLEX2 1.5[5] by dual-space direct methods with SHELXT,[6] by full-matrix least-squares refinement using SHELXL-2014/7.[7] All non-hydrogen atoms were refined anisotropically. The contribution of the hydrogen atoms, in their calculated positions, was included in the refinement using a riding model. A full listing of atomic coordinates, bond lengths, angles and displacement parameters for all the structures have been deposited at the Cambridge Crystallographic Data Centre. For the individual numbers, please refer to the XRD table in section 6.

1.14 UV/VIS measurements of dibenzyl azodicarboxylate

Absorption spectra were recorded using a CARY 3500 UV/VIS spectrometer by Agilent.

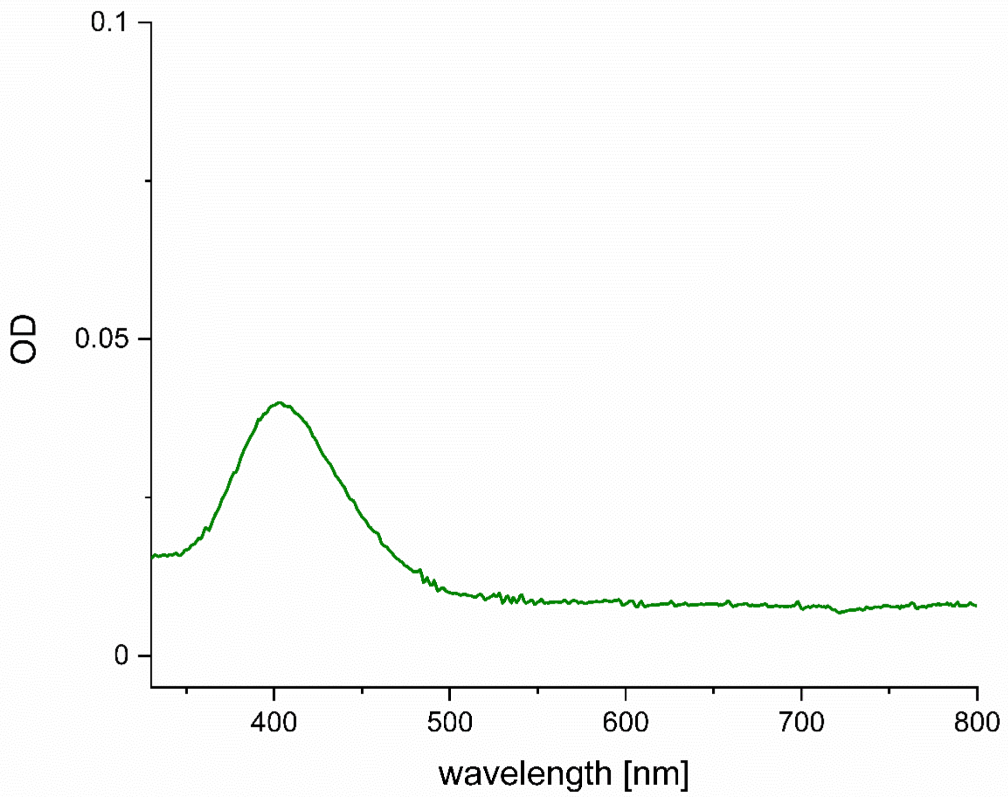

Figure S3. Absorption spectrum of DBAD in acetone.

2 Literature reports on terpene-derived diamines

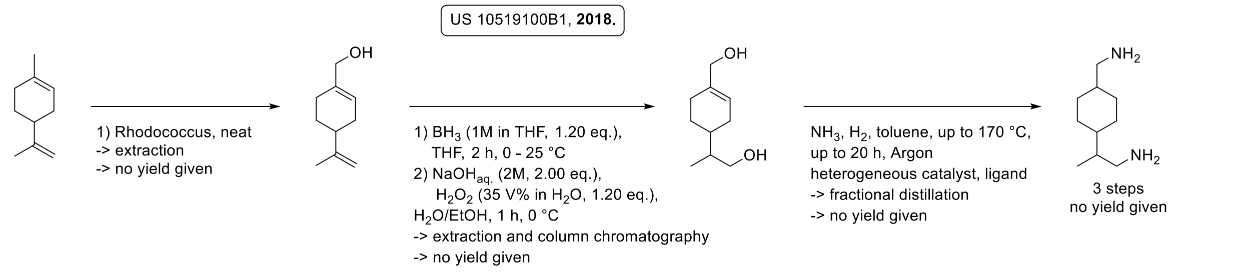

Figure L1. Patented synthesis of 0,10-*p*-menthane diamine.[8]

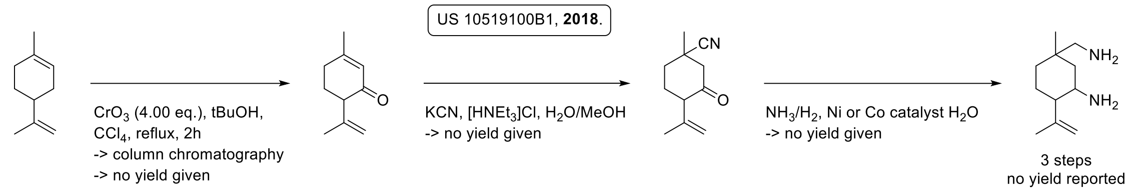

Figure L2. Patented synthesis of 1,3-*p*-menthene diamine.[8]

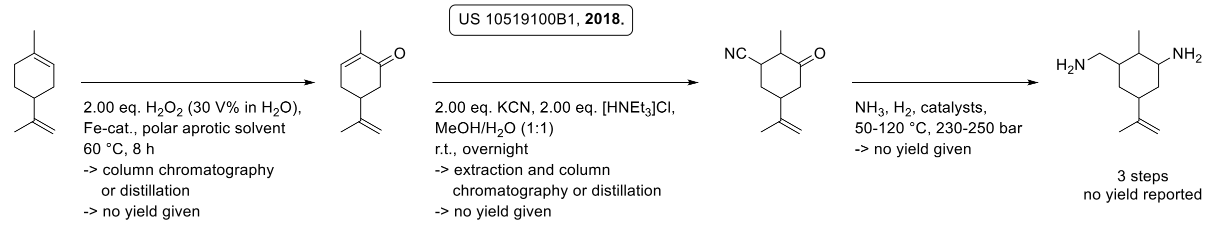

Figure L3. Patented synthesis of 2,2'-*p*-menthene diamine.[8]

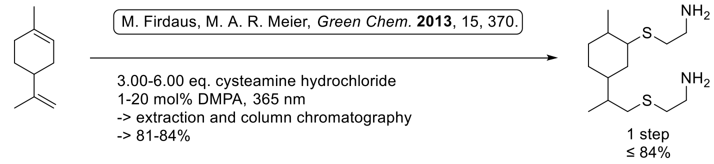

Figure L4. Published synthesis of *p*-menthane based diamine by thiol-ene reaction of limonene.[9]

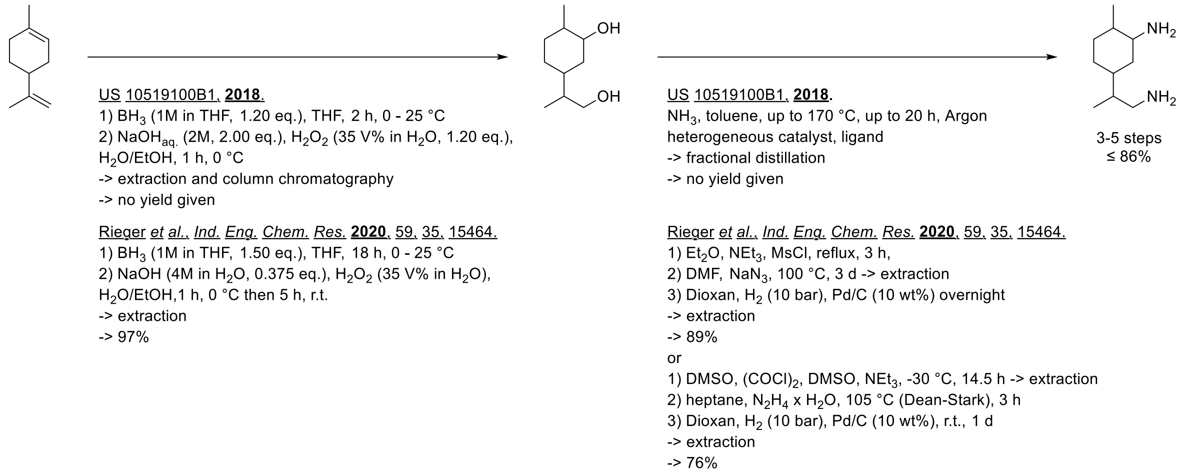

Figure L5. Published and patented syntheses of 2,9-*p*-menthane diamine.[8,10]

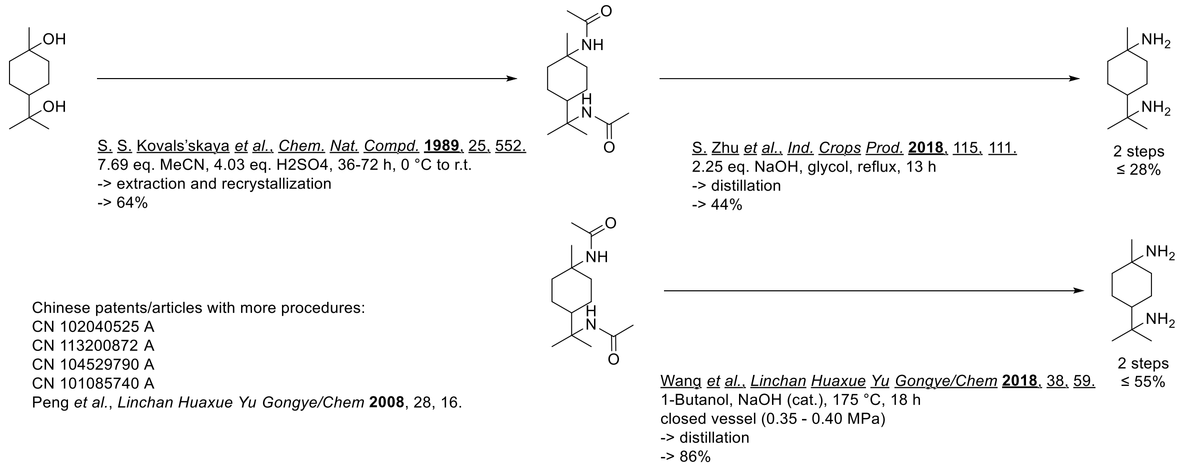

Figure L6. Published and patented syntheses of 1,8-p-menthane diamine from limonene diol.[11]

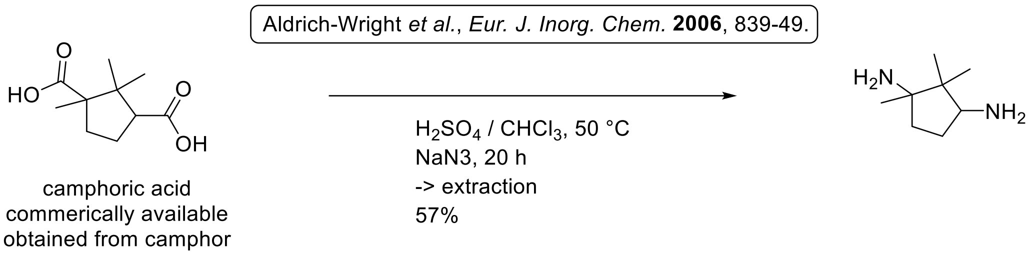

Figure L7. Published synthesis of campor-derived 1,3-cyclopentane diamine.[12]

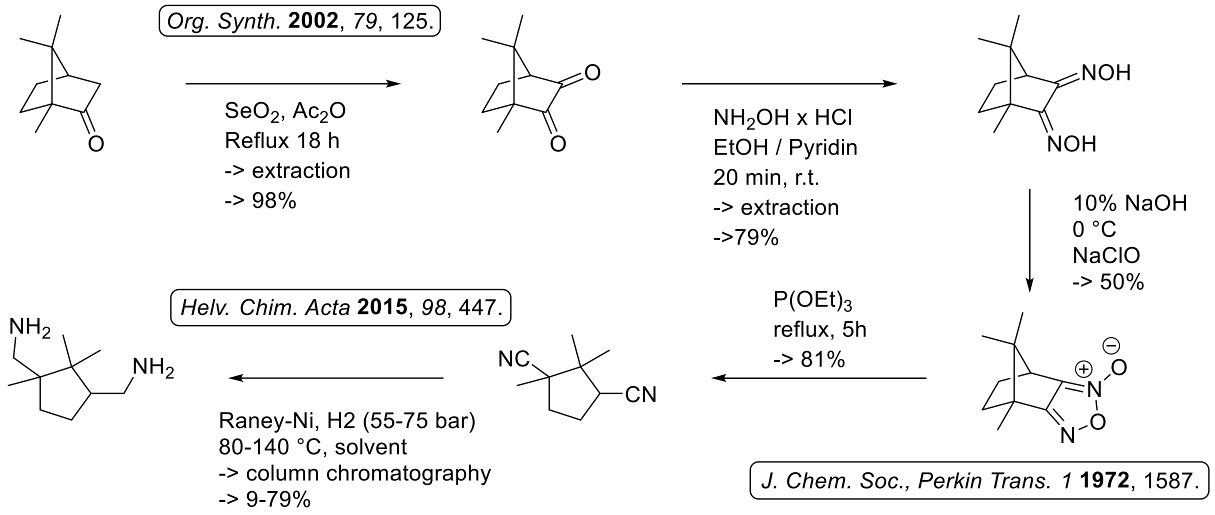

Figure L8. Synthesis of camphor-derived bis(aminomethylen) cyclopentane.[13]

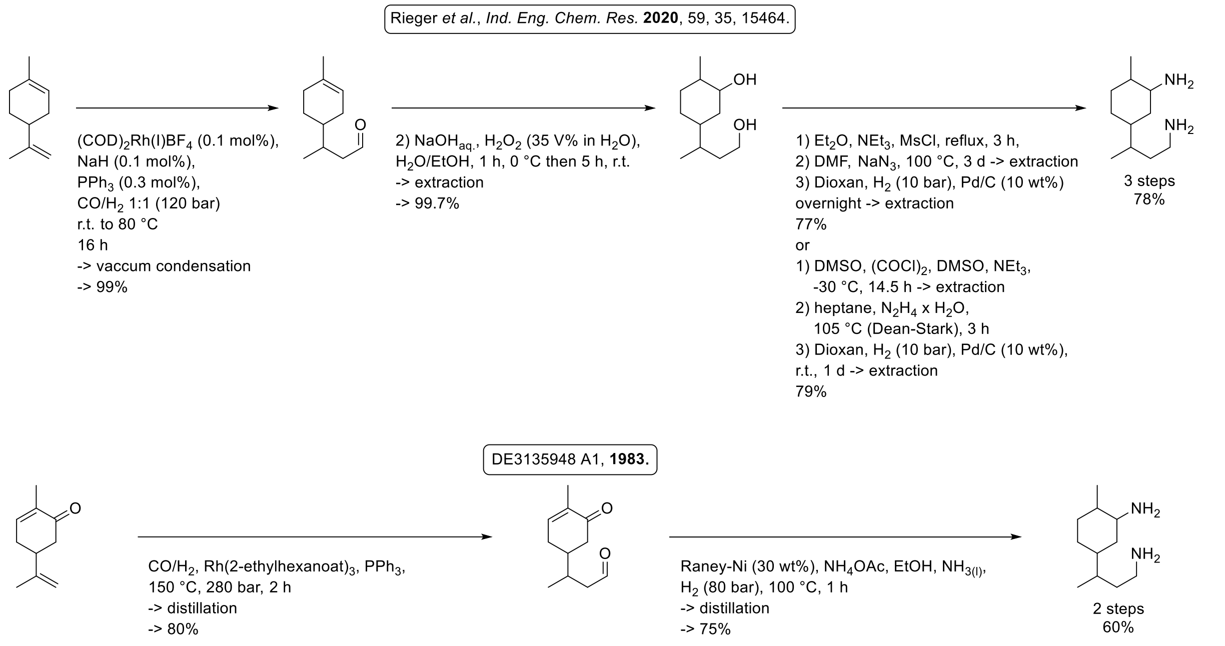

Figure L9. Published (top)[10] and patented (bottom)[14] syntheses of 2,11-*p*-menthane diamine.

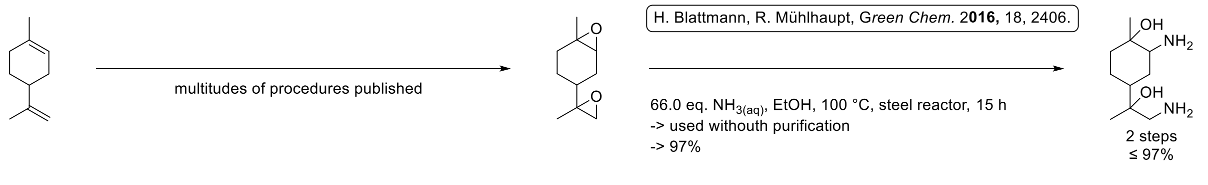

Figure L10. Published synthesis of *p*-menthane based bis(amino alcohol).[15]

3 Optimization of the di-aza-Diels-Alder reaction of 1 with DBAD

3.1 Alder-Ene reaction as competing thermal reaction

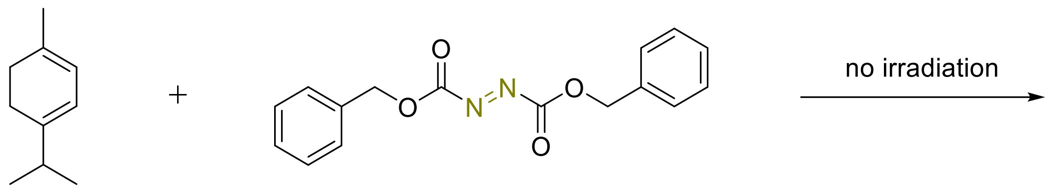

- Test reaction of **1** with DBAD neat without irradiation:

10.4 µL of **1** (73% pure, 6.37 mg, 46.8 µmol, 1.00 equiv.) were mixed with 14.9 mg DBAD (96%, 48.0 µmol, 1.02 equiv.) and retained unstirred for 60 minutes. The mixture was dissolved in 0.5 mL acetone-d_6 and anisole was added for external standardization.

NMR-yield of **2a**: 7%

- GC kinetic of reaction of **1** with DBAD in solution without irradiation:

209 µL of **1** (73% pure, 128 mg, 941 µmol, 1.00 equiv.) and 298 mg DBAD (96% pure, 959 µmol, 1.02 equiv.) were dissolved in 2.00 mL acetone (0.47 mol/L) and tetradecane was added for internal standardization. The mixture was stirred for 18.5 hours and the conversion of **1** was monitored by GC.

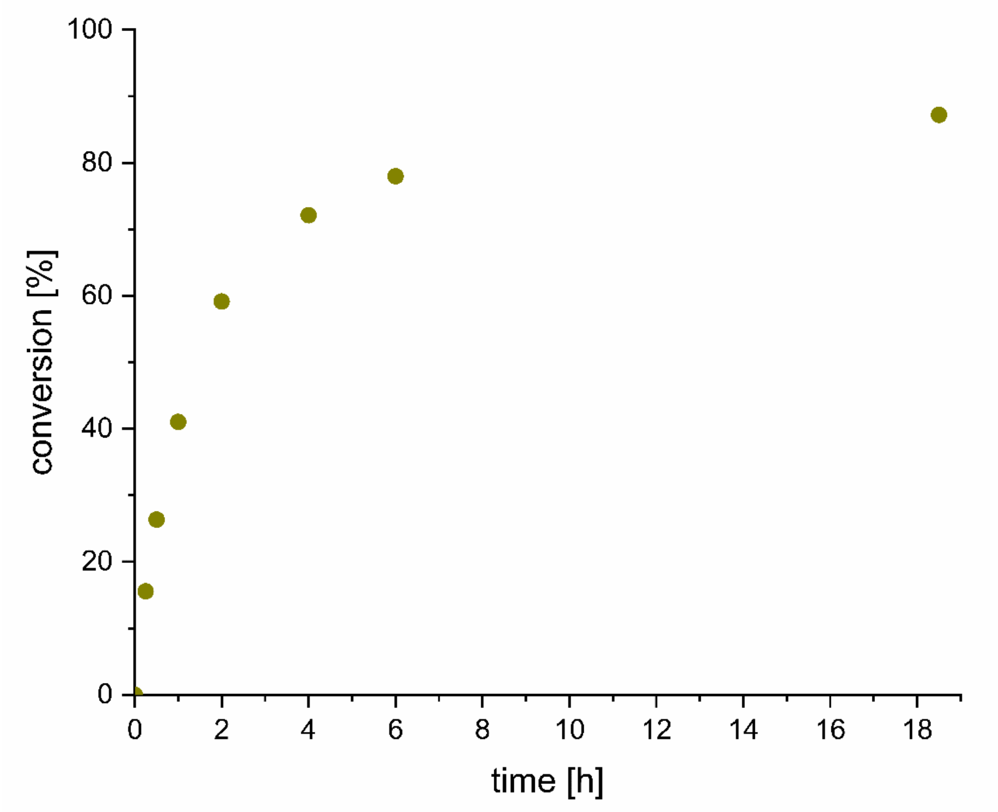

Figure S4. GC conversion of **1** during the black reaction with DBAD.

3.2 Screenings for optimizing the di-aza-*Diels-Alder* reaction

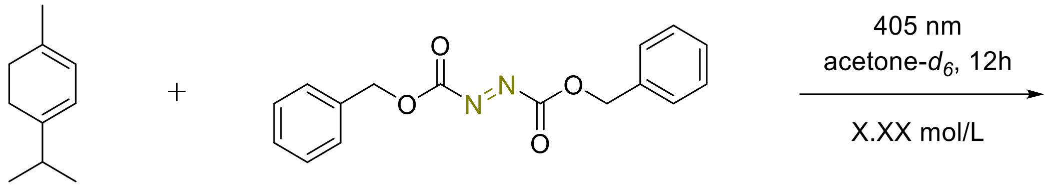

- Concentration screening of photochemical reaction between **1** and DBAD:

XX.X µL of 1 (73% pure, XX.X mg, XX.X µmol, 1.00 equiv.) and XX.X mg DBAD (96% pure, XX.X µmol, 1.02 equiv.), all exact amounts of individual reactions are given in Table S1, were dissolved in 0.5 mL acetone-d_6 and irradiated unstirred within an NMR tube for 12 hours. Prior to ^{1}H NMR measurements, anisole was added for standardization.

Table S1. ^{1}H NMR yields of photo reaction between **1** and DBAD at varied concentrations.

V (**1**) [µL]	m (**1**) [mg]	n (**1**) [µmol]	m (DBAD) [mg]	n (DBAD) [µmol]	c [mol/L]	**NMR yield [%]**
2.60	1.59	11.7	3.73	12.0	0.02	23
5.20	3.19	23.4	7.46	24.0	0.05	34
7.80	4.78	35.1	11.2	36.0	0.07	44
10.4	6.38	46.8	14.9	48.0	0.09	52
20.8	12.8	94.0	29.8	96.0	0.19	**57**
52.0	31.9	234	74.6	240	0.47	51

3 Optimization of the di-aza-Diels-Alder reaction of 1 with DBAD

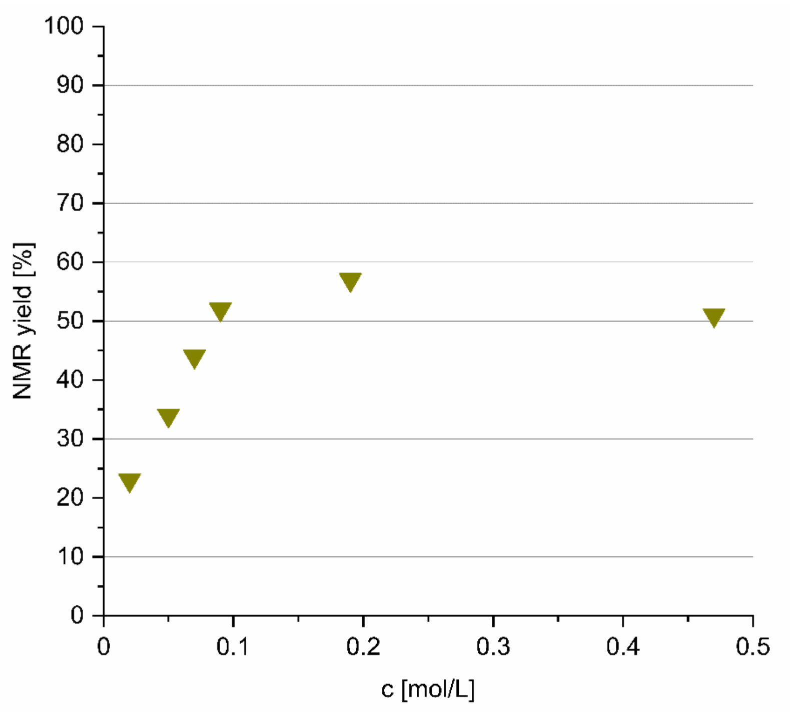

Figure S5. ^{1}H NMR yields of photo reaction between **1** and DBAD at varied concentrations.

3 Optimization of the di-aza-Diels-Alder reaction of 1 with DBAD

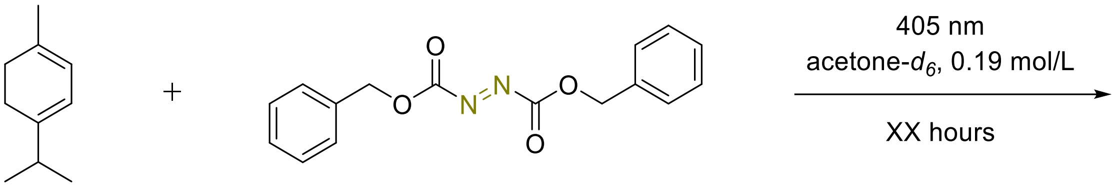

➢ Time screening of photochemical reaction between **1** and DBAD:

20.8 µL of 1 (73% pure, 12.8 mg, 94.0 µmol, 1.00 equiv.) and 29.8 mg DBAD (96% pure, 96.0 µmol, 1.02 equiv.) were dissolved in 0.5 mL acetone-d_6 and anisole was added for standardization. The mixtures were irradiated unstirred within an NMR tube for varied amounts of time.

Table S2. ^{1}H NMR yields of time screening of photochemical reaction between **1** and DBAD.

Reaction time [h]	NMR yield [%]
0.25	22
0.50	38
0.75	44
1.00	46
2.00	**57**
3.00	57
4.00	58
12.0	54

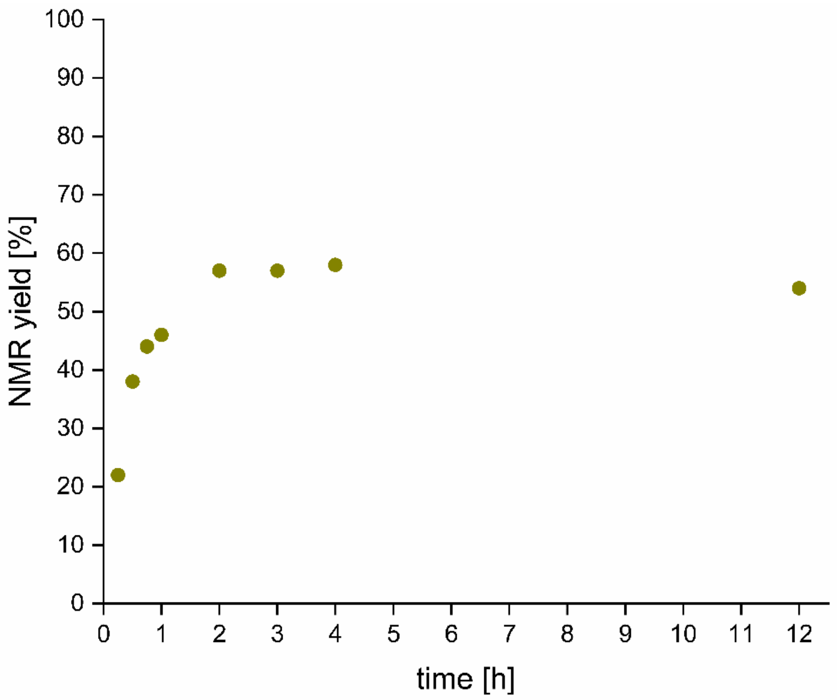

Figure S6. ^{1}H NMR yields of time screening of photochemical reaction between **1** and DBAD.

3 Optimization of the di-aza-Diels-Alder reaction of 1 with DBAD

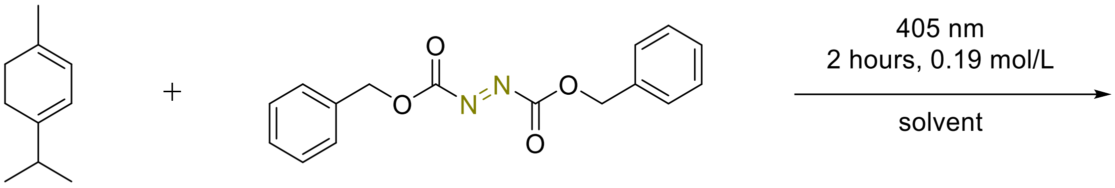

- Solvent screening of photochemical reaction between **1** and DBAD:

20.8 µL of **1** (73% pure, 12.8 mg, 94.0 µmol, 1.00 equiv.) and 29.8 mg DBAD (96% pure, 96.0 µmol, 1.02 equiv.) were dissolved in 0.5 mL of varied solvents. The mixtures were irradiated while stirring in 5 mL headspace flasks for 2 hours. Afterwards, the solvent was evaporated under reduced pressure and the residues were dissolved in 0.5 mL acetone-d_6. Anisole was added for standardization and the mixtures were investigated by NMR spectroscopy.

In the case of DMSO, the reaction and ^{1}H NMR measurements were both directly conducted in DMSO-d_6.

Table S3. ^{1}H NMR yields of solvent screening of photochemical reaction between **1** and DBAD.

solvent	NMR yield [%]	solvent	NMR yield [%]
n-heptane	54	acetone	58
cyclohexane	**71**	CH_2Cl_2	47
toluene	67	$CHCl_3$	43
Et_2O	65	*t*BuOH	54
iPr_2O	**69**	*i*PrOH	49
THF	57	EtOH	43
2-Me-THF	56	MeOH	24
EtOAc	63	MeCN	44
*t*BuOAc	52	DMSO-d_6	22
DMC	60	H_2O	22

4 Spectra

4.1 NMR spectra

Dibenzyl (1R,4R)-1-isopropyl-4-methyl-2,3-diazabicyclo[2.2.2]oct-5-ene-2,3-dicarboxylate (**2**)

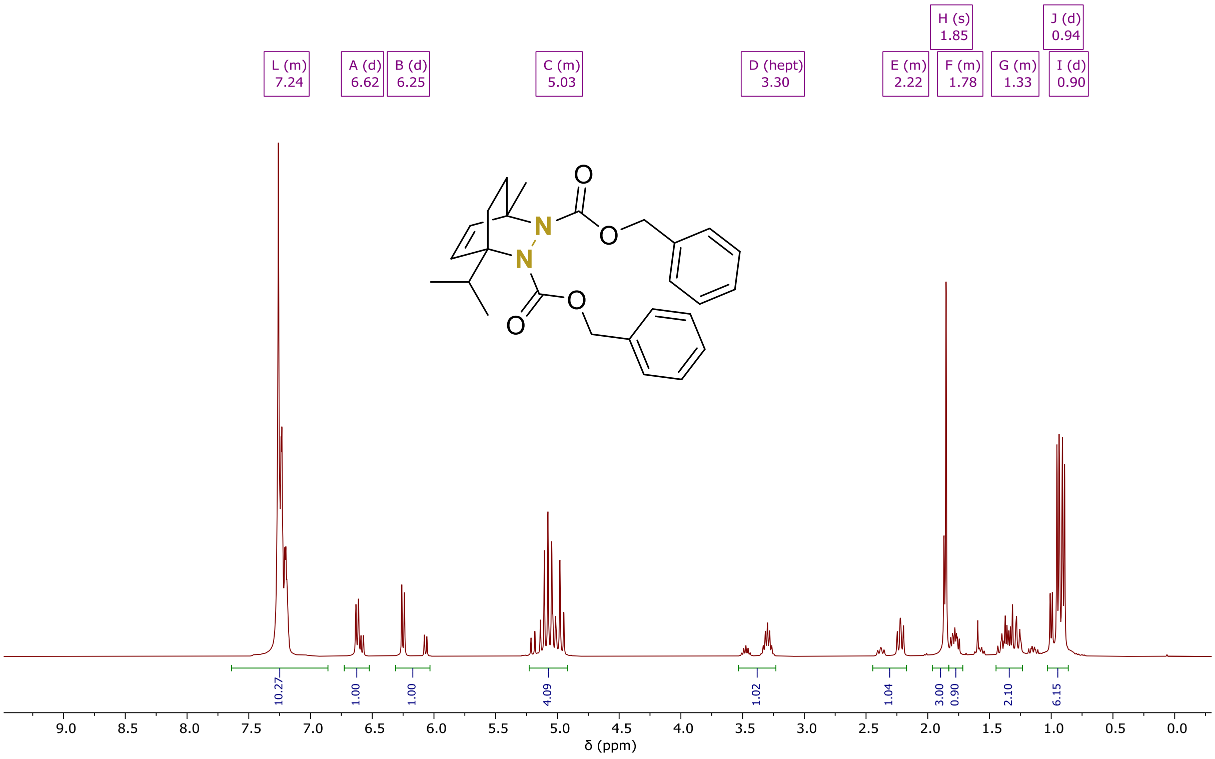

Figure S7. ^{1}H NMR spectrum of **2a** in $CDCl_3$.

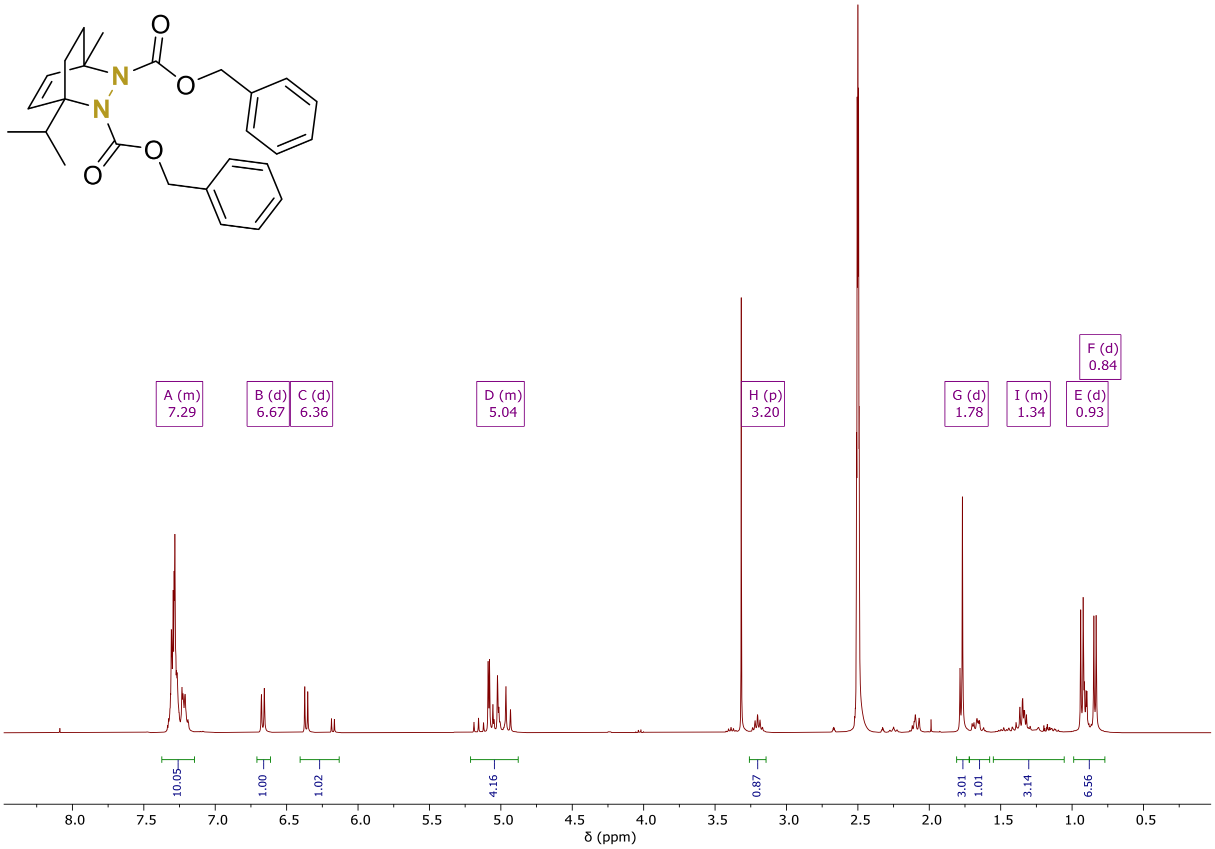

Figure S8. ^{1}H NMR spectrum of **2a** in DMSO-d_6.

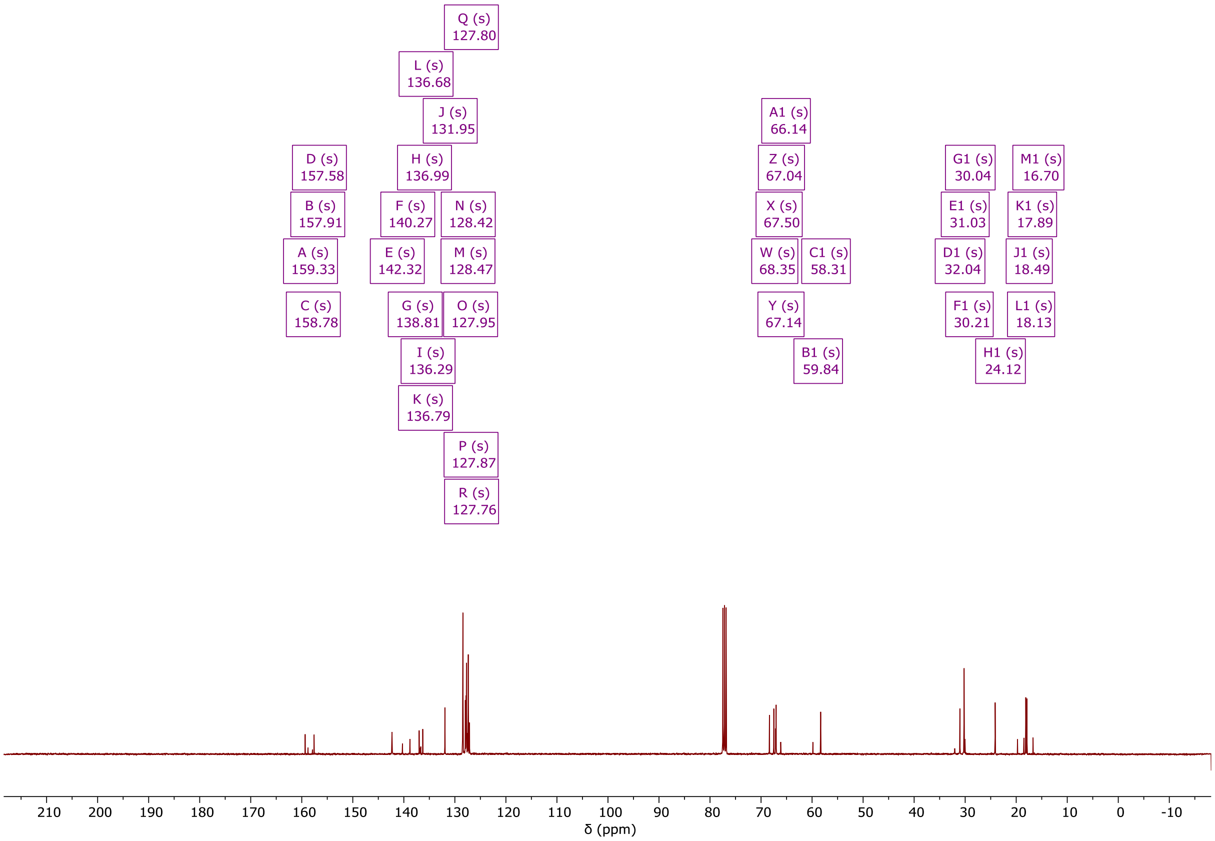

Figure S9. ^{13}C NMR spectrum of **2a** in $CDCl_3$.

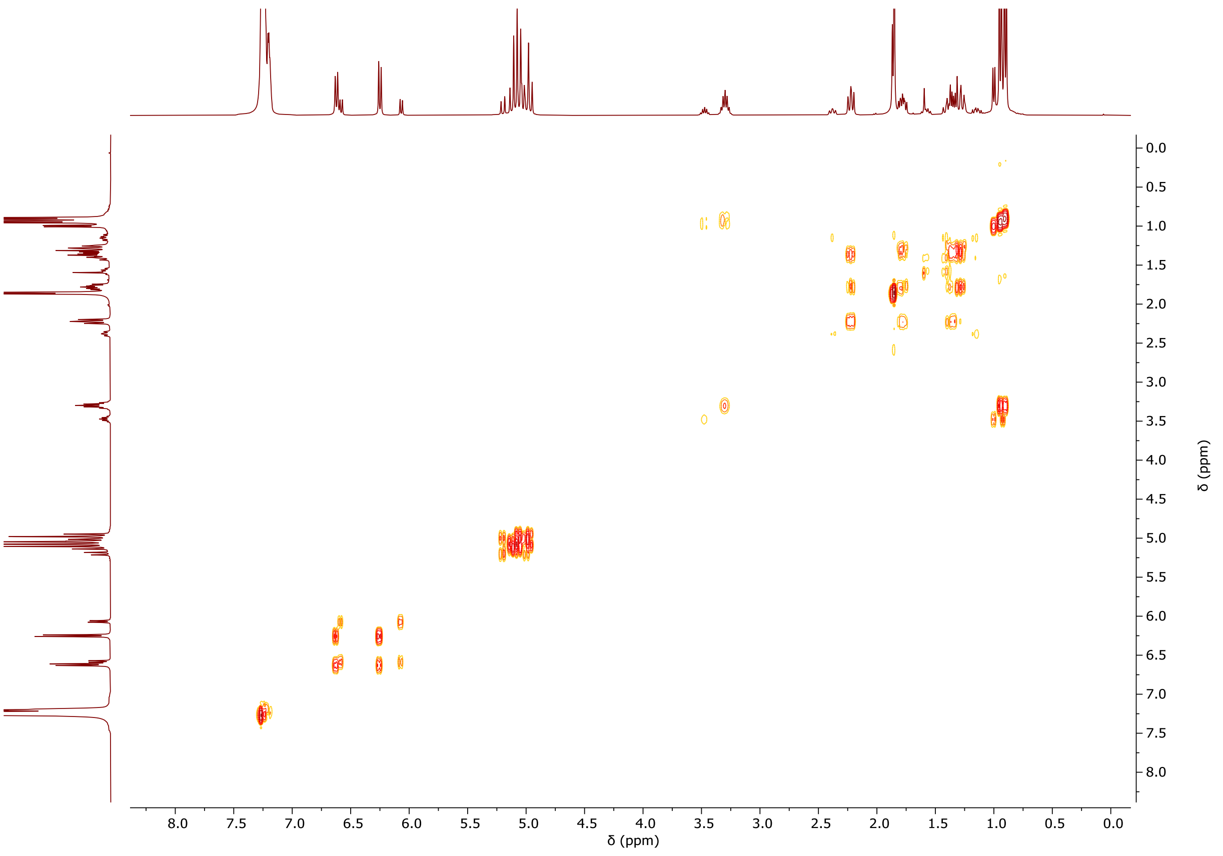

Figure S10. $^{1}H^{1}H$ gCOSY NMR spectrum of **2a** in $CDCl_3$.

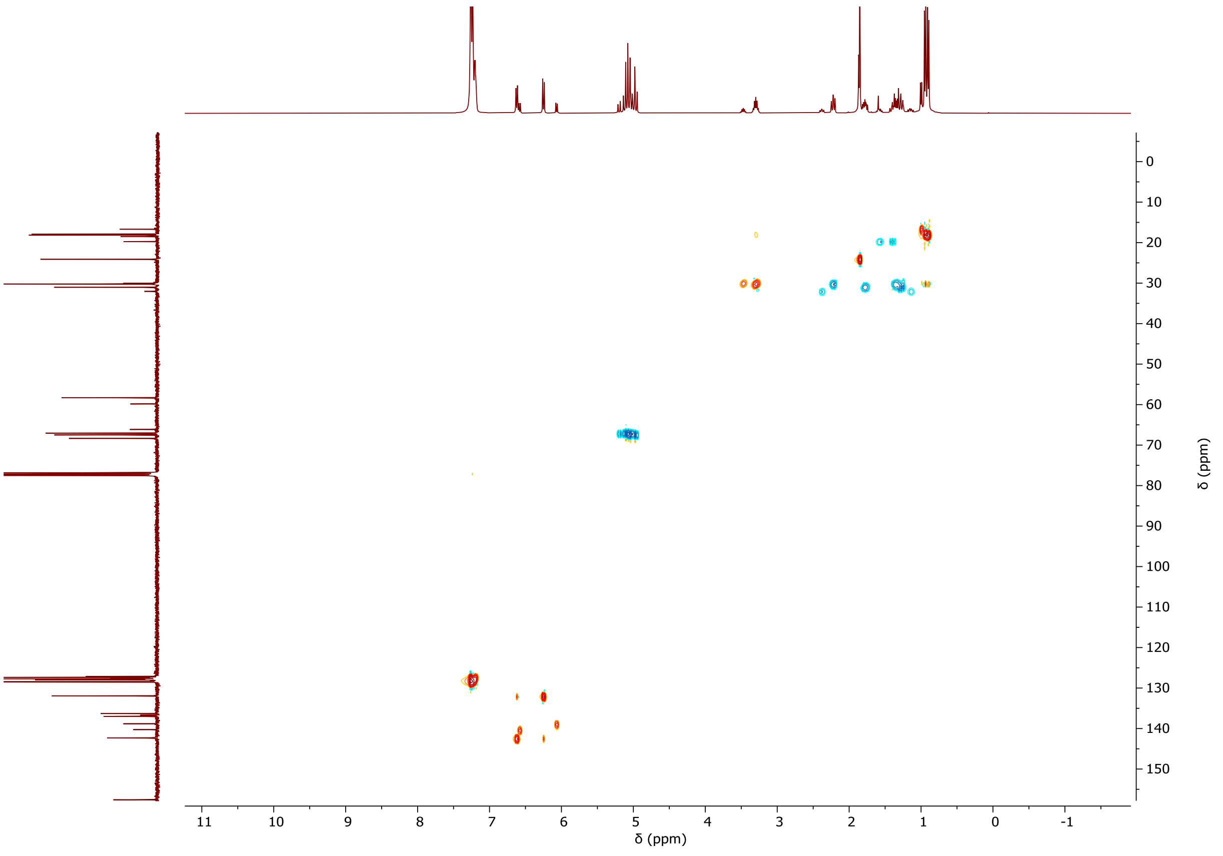

Figure S11. $^{1}H^{13}C$ $HSQC_{ed}$ NMR spectrum of **2a** in $CDCl_3$.

4 Spectra

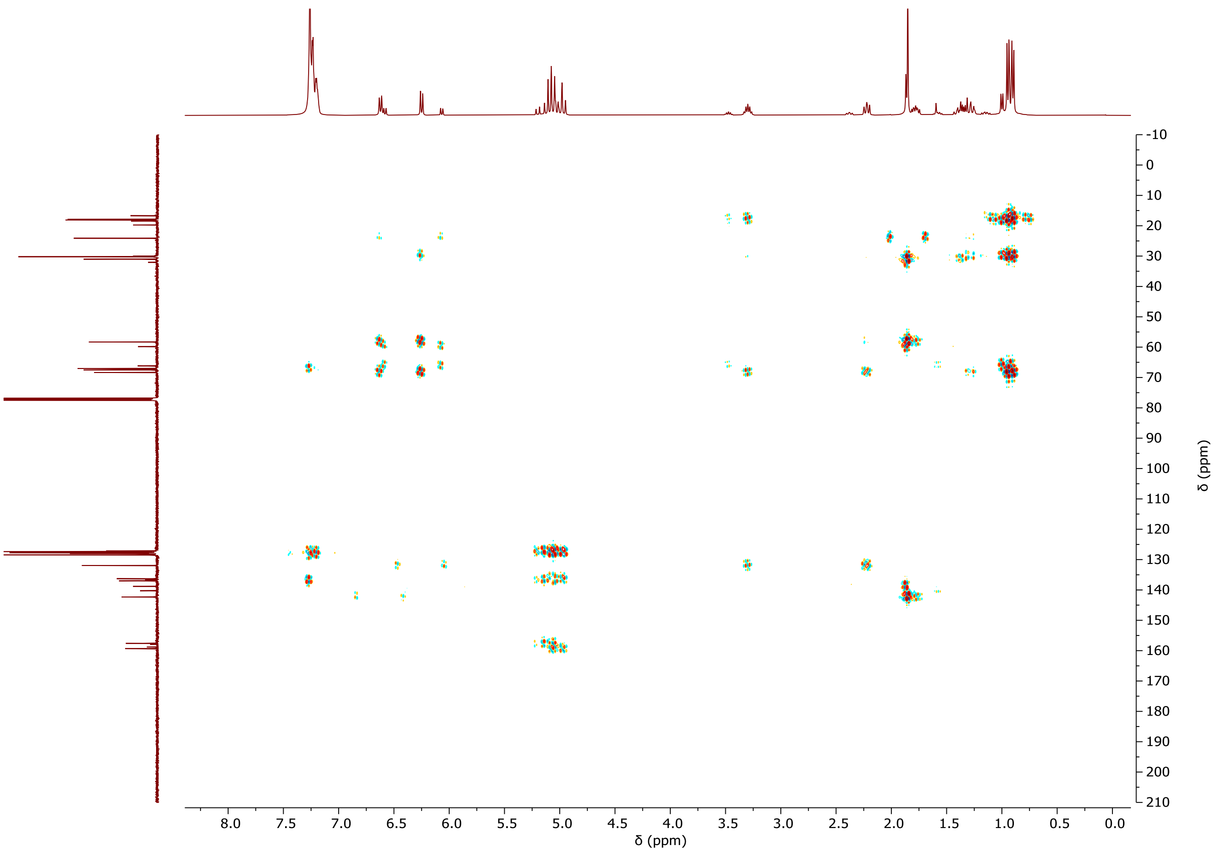

Figure S12. $^{1}H^{13}C$ HMBC NMR spectrum of **2a** in $CDCl_3$.

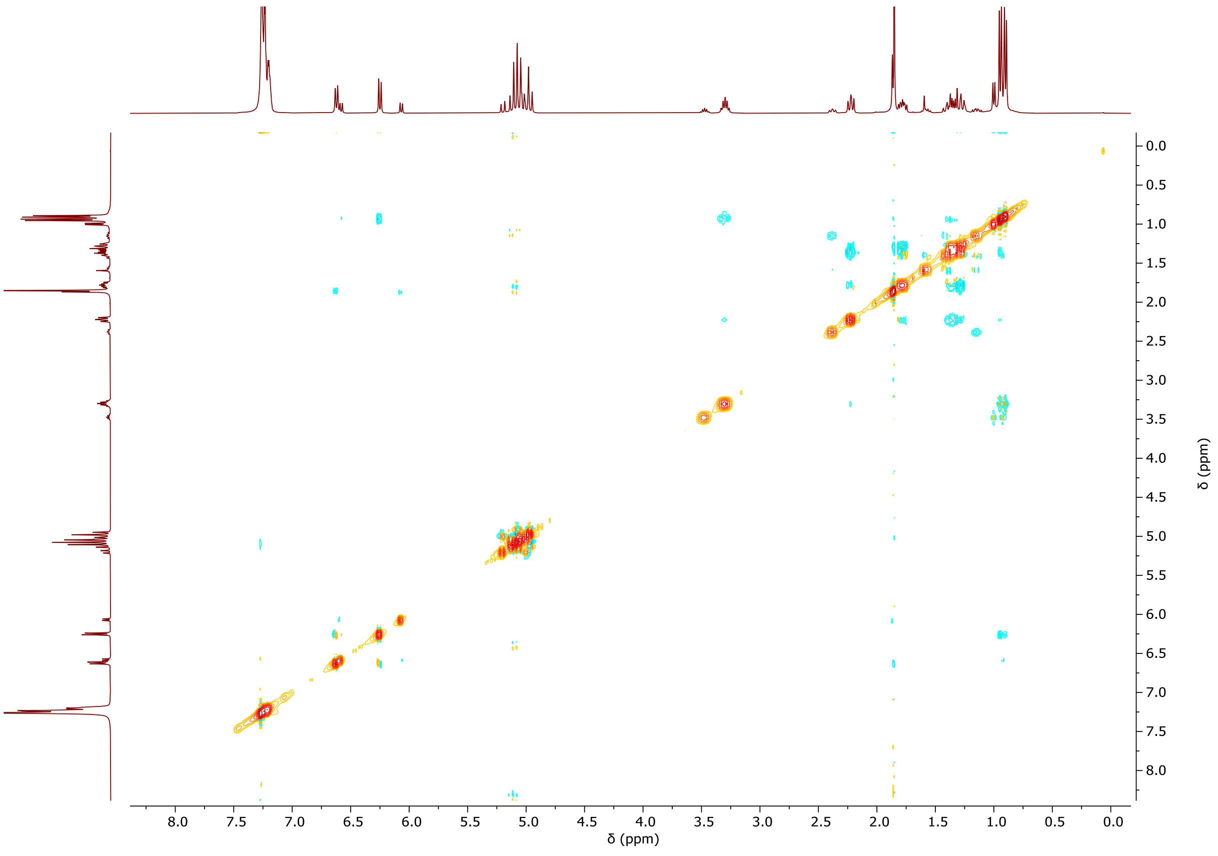

Figure S13. $^{1}H^{1}H$ NOESY NMR spectrum of **2a** in $CDCl_3$.

Dibenzyl 1-(5-isopropyl-2-methylcyclohexa-2,5-dien-1-yl)hydrazine-1,2-dicarboxylate (**2b**):

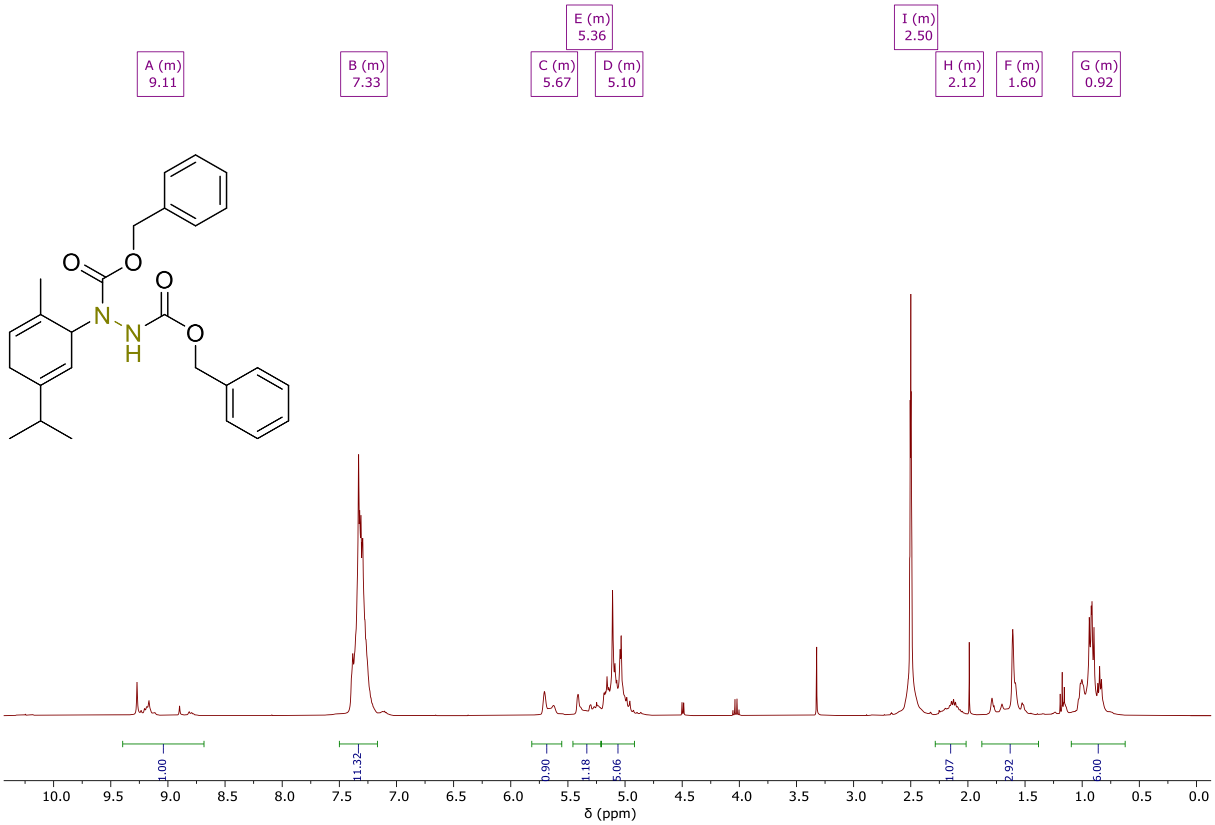

Figure S14. ^{1}H NMR spectrum of **2b** in DMSO-d_6.

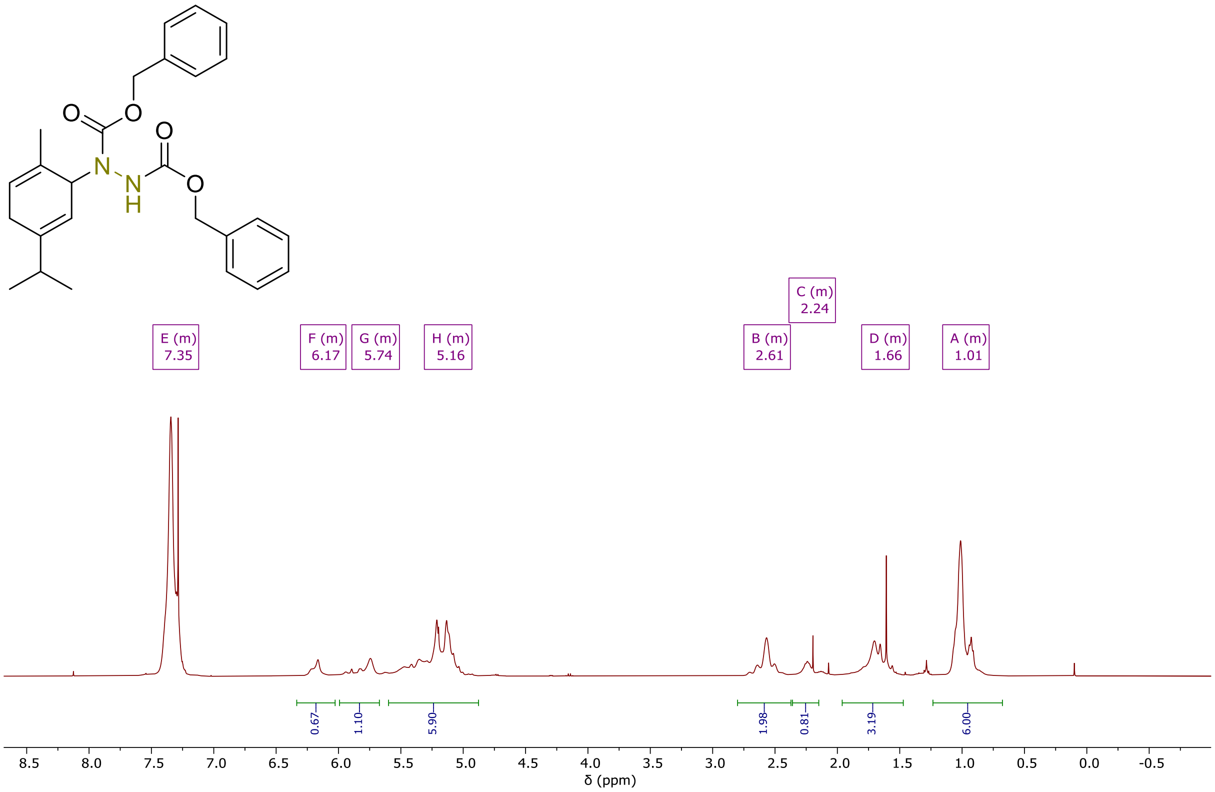

Figure S15. ^{1}H NMR spectrum of **2b** in $CDCl_3$.

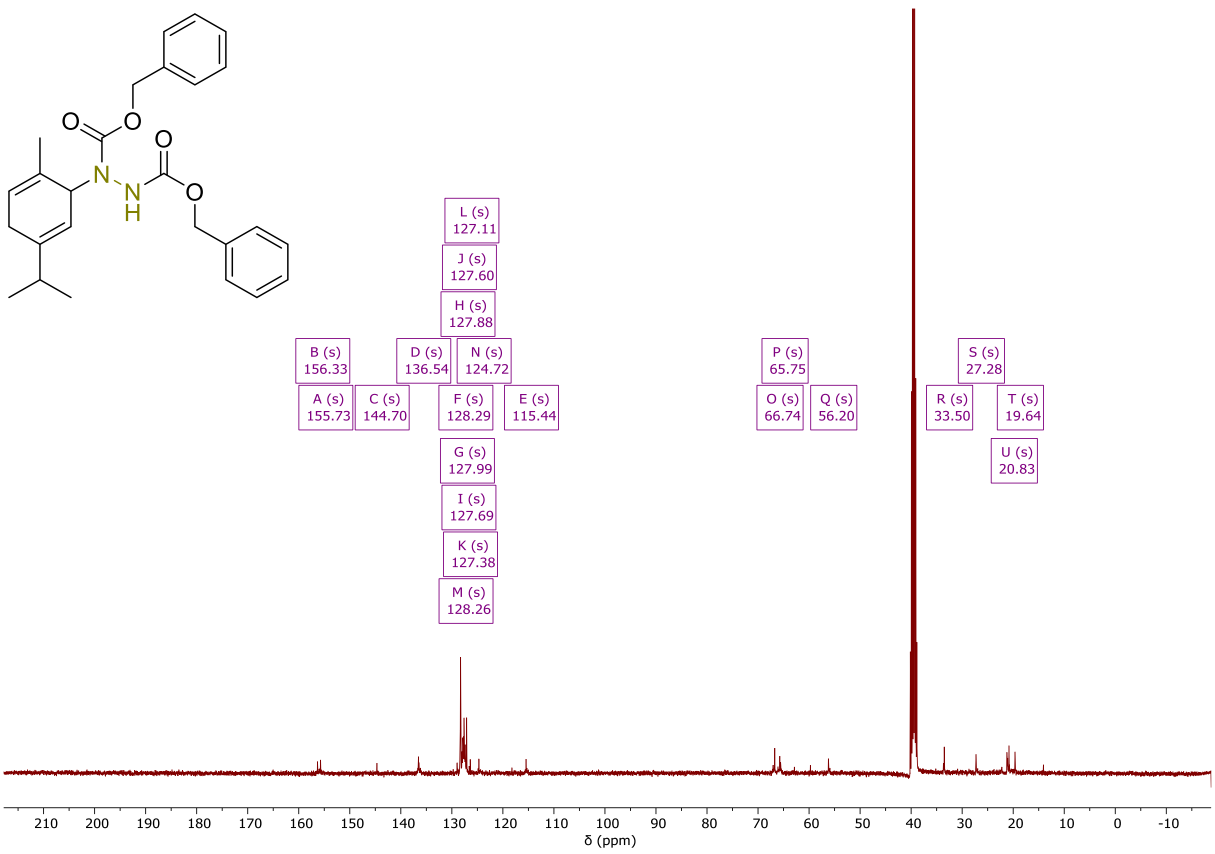

Figure S16. ^{13}C NMR spectrum of **2b** in DMSO-d_6.

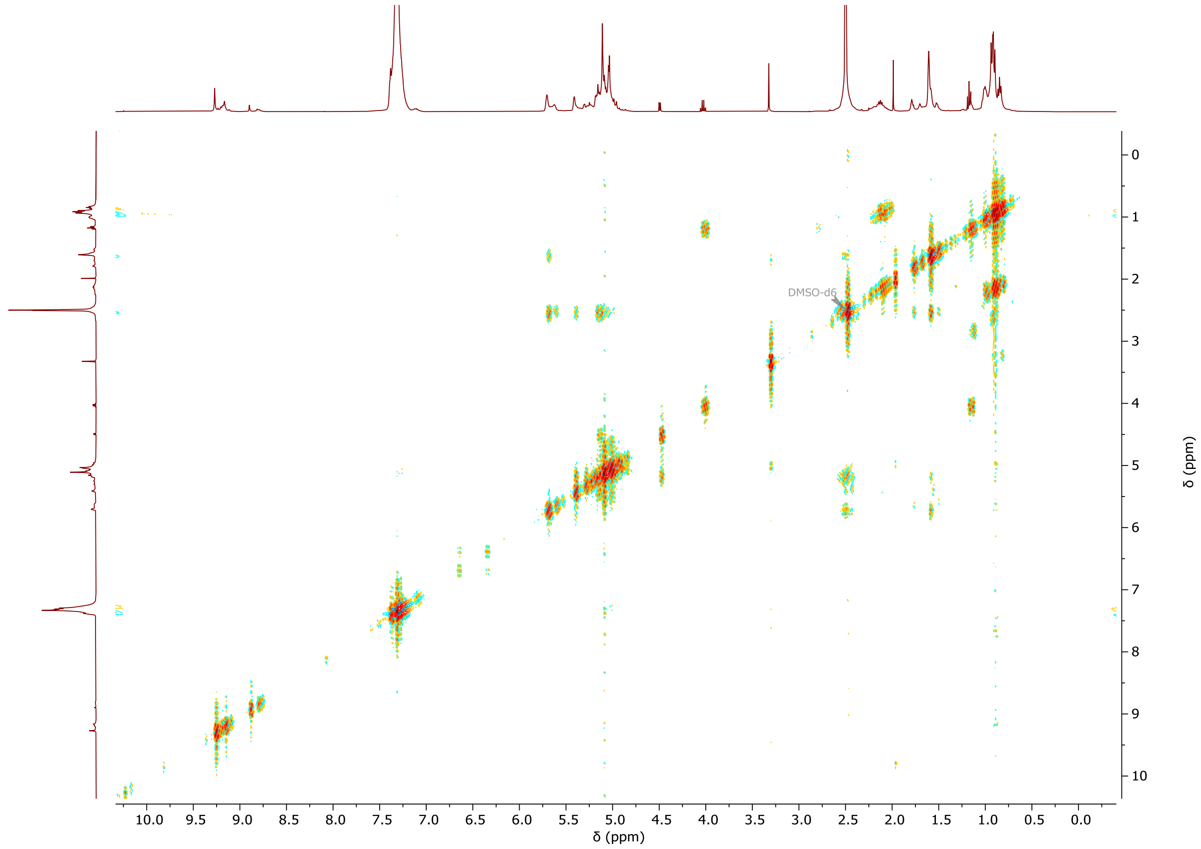

Figure S17. ^{1}H^{1}H gCOSY NMR spectrum of **2b** in DMSO-d_6.

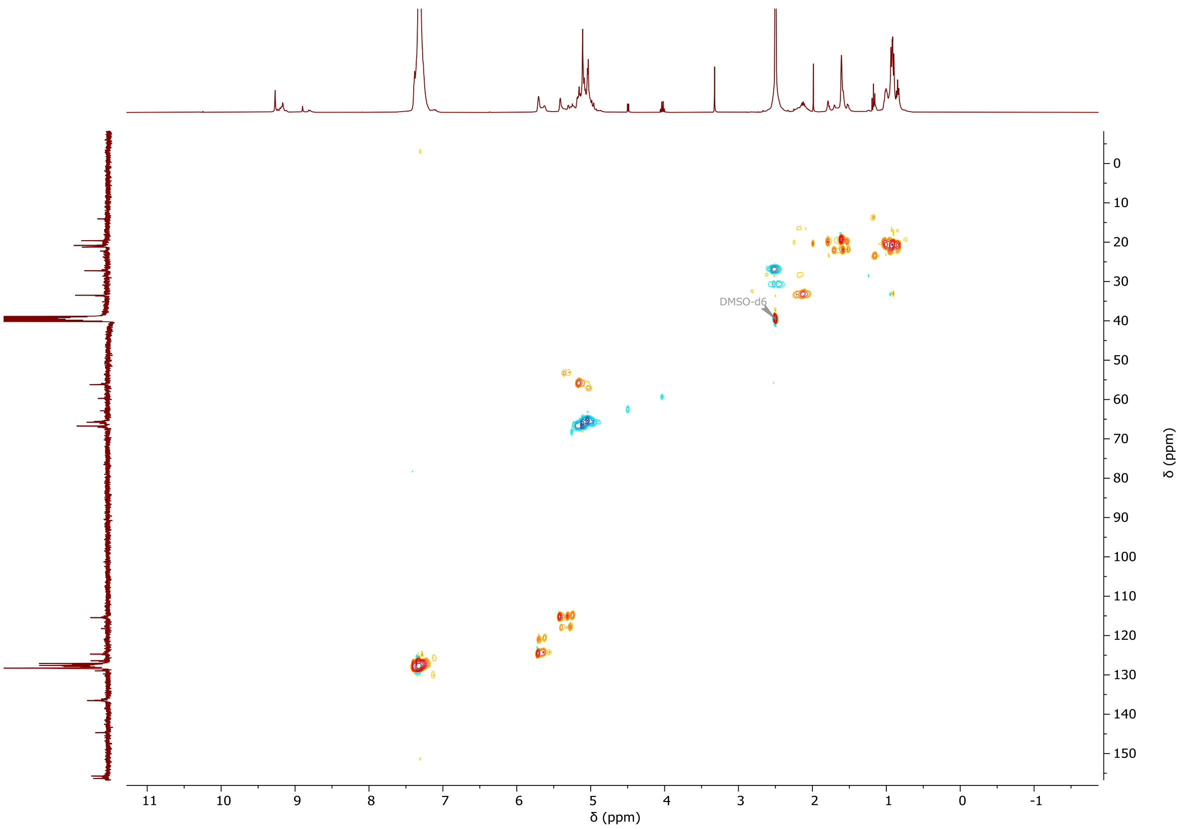

Figure S18. $^{1}H^{13}C$ HSQC$_{ed}$ NMR spectrum of **2b** in DMSO-*d*$_6$.

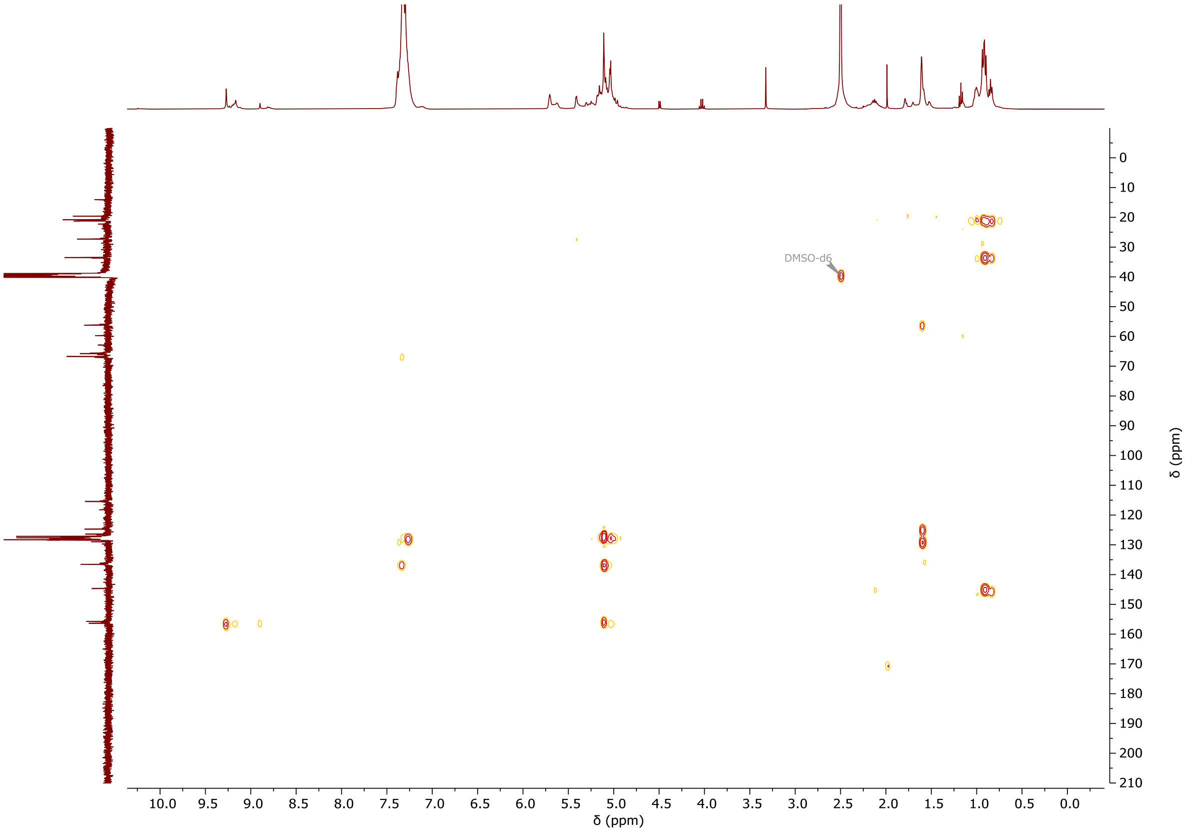

Figure S19. $^{1}H^{13}C$ HMBC NMR spectrum of **2b** in DMSO-*d*$_6$.

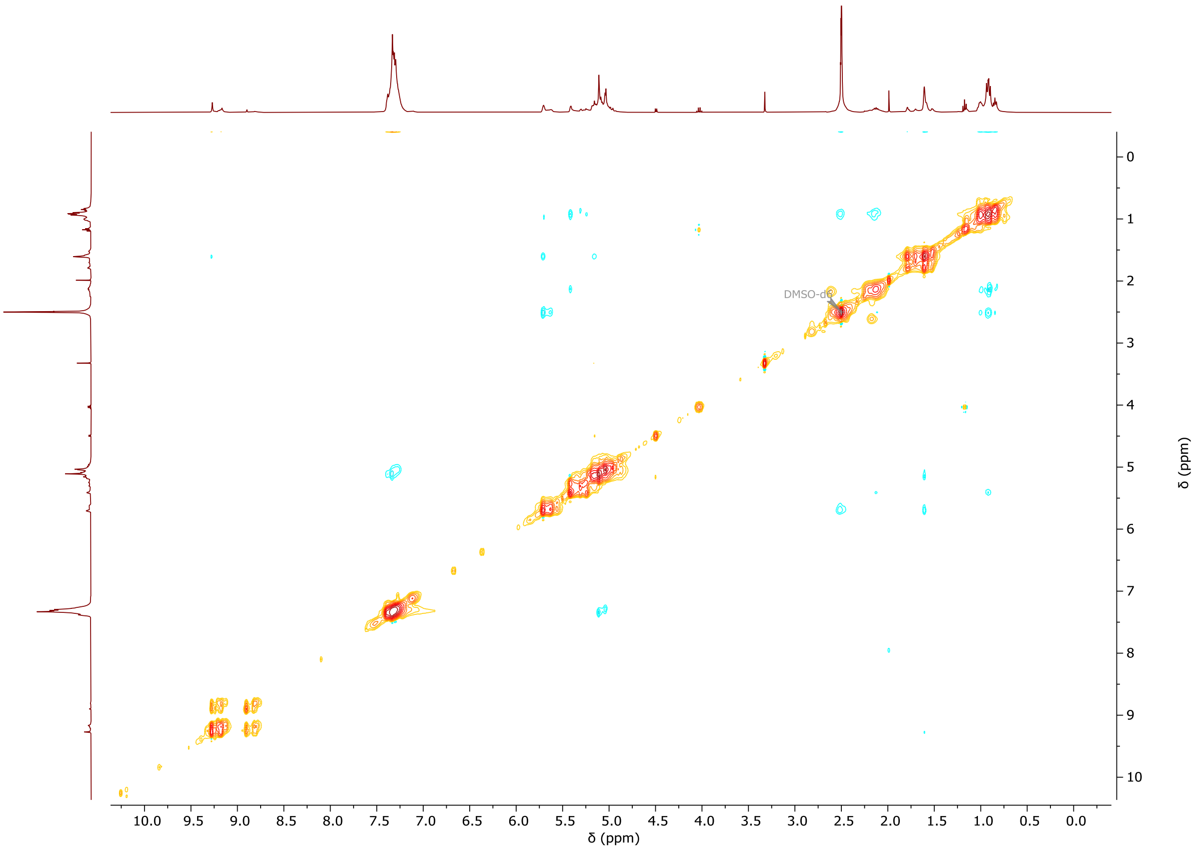

Figure S20. $^1H^1H$ NOESY NMR spectrum of **2b** in DMSO-d_6.

1,4-*p*-Menthane diamine (**3**)

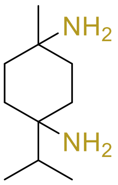

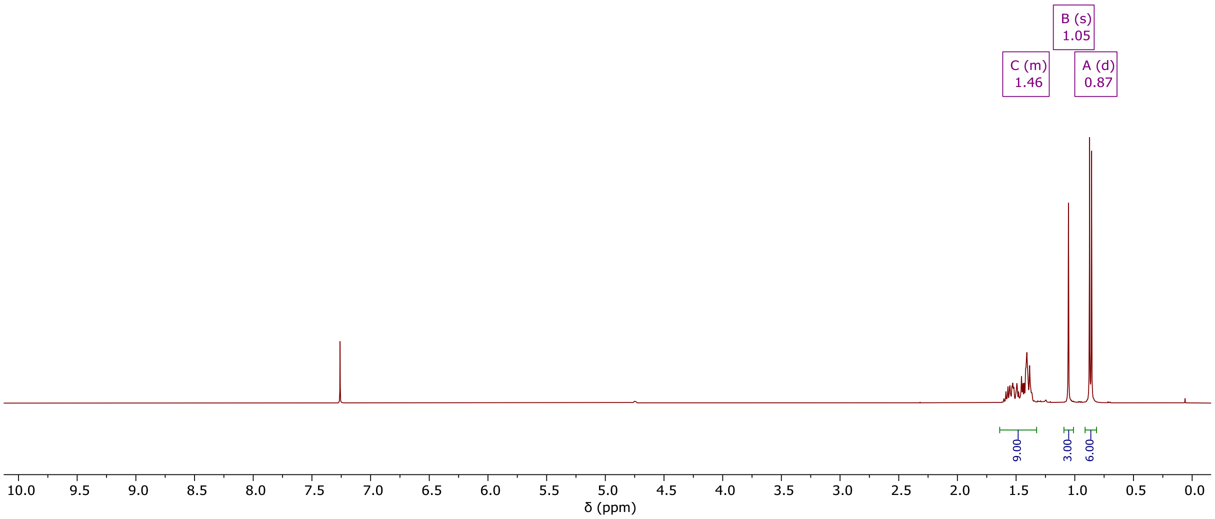

Figure S21. ^{1}H NMR spectrum of **3** in $CDCl_3$.

4 Spectra

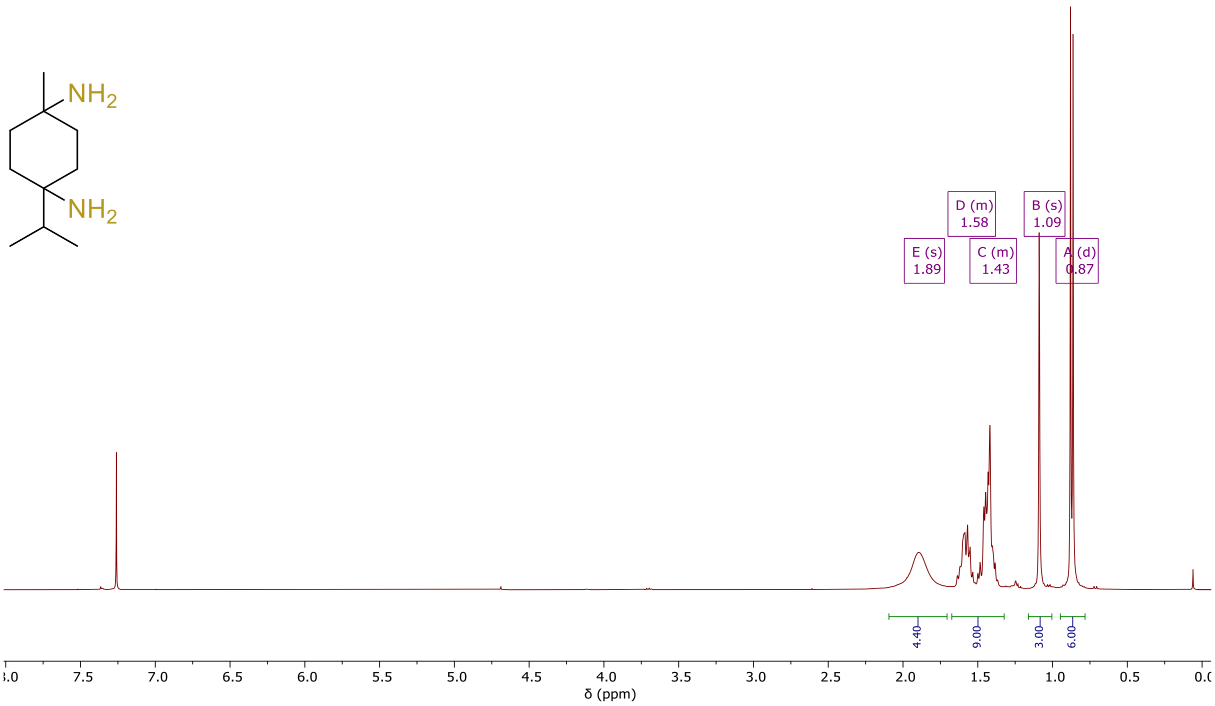

Figure S22. ^{1}H NMR spectrum of crude **3** in $CDCl_3$.

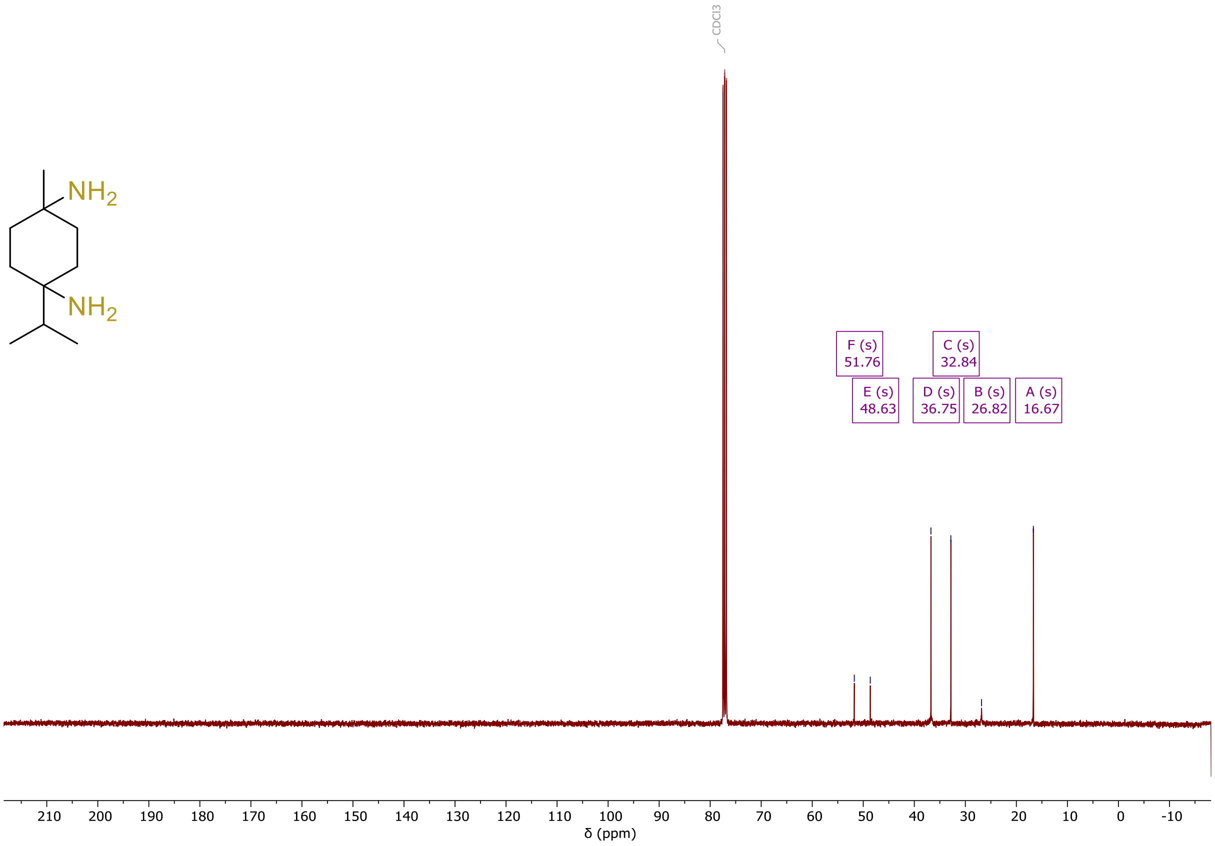

Figure S23. ^{13}C NMR spectrum of **3** in $CDCl_3$.

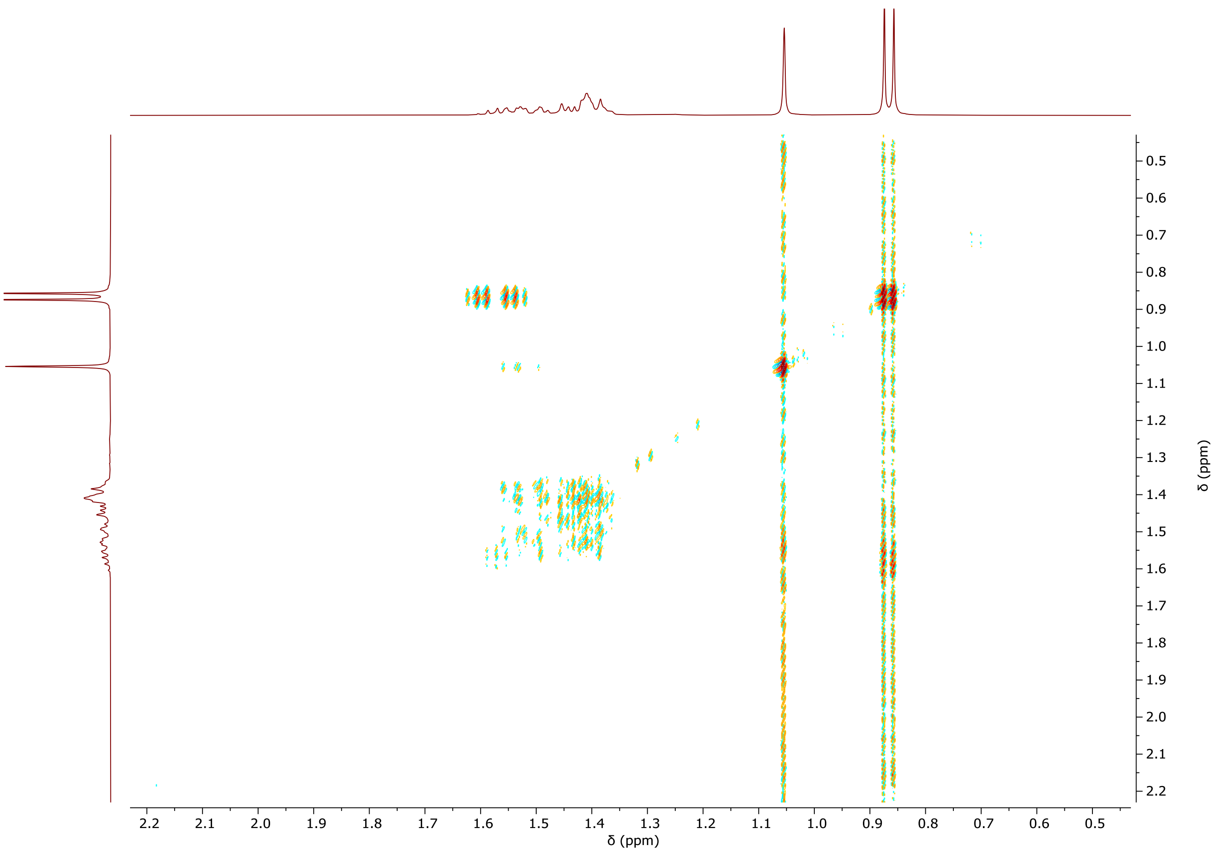

Figure S24. $^{1}H^{1}H$ gCOSY NMR spectrum of **3** in $CDCl_3$.

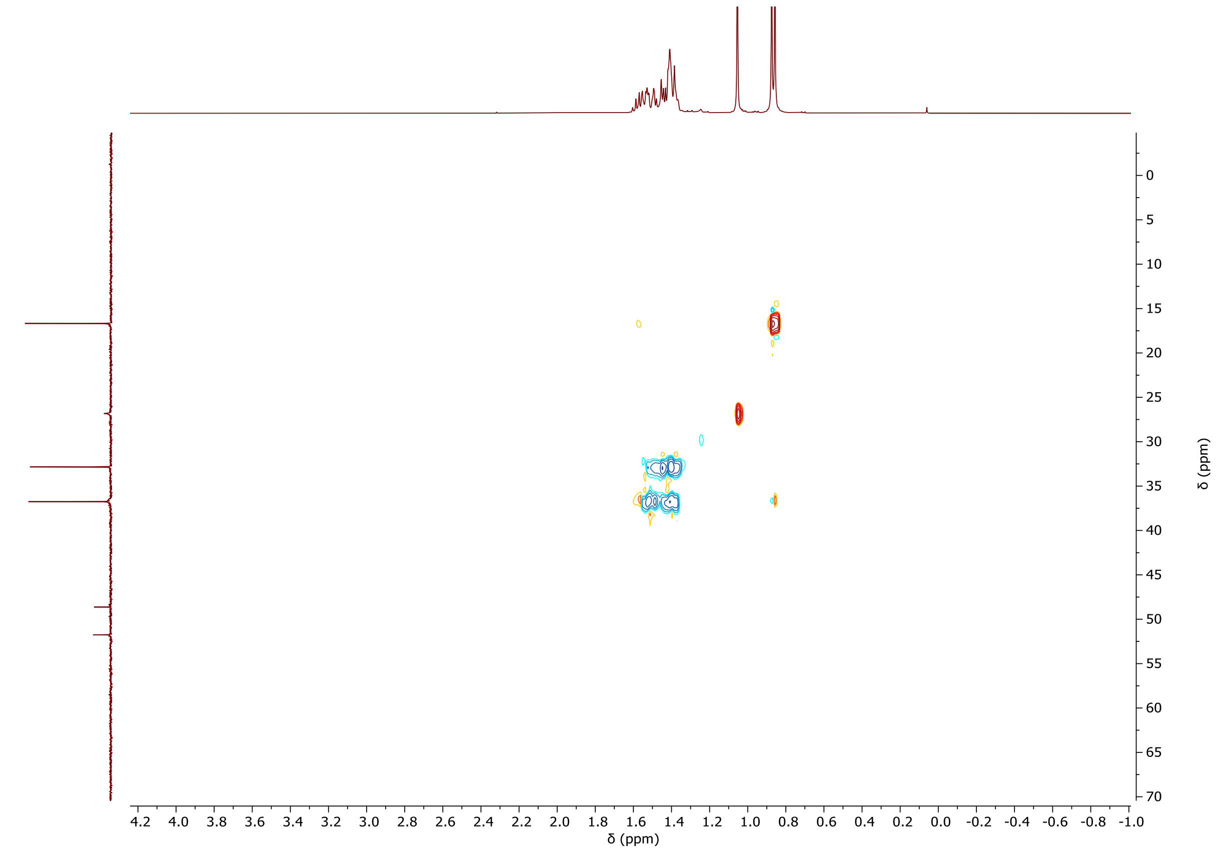

Figure S25. $^{1}H^{13}C$ $HSQC_{ed}$ NMR spectrum of **3** in $CDCl_3$.

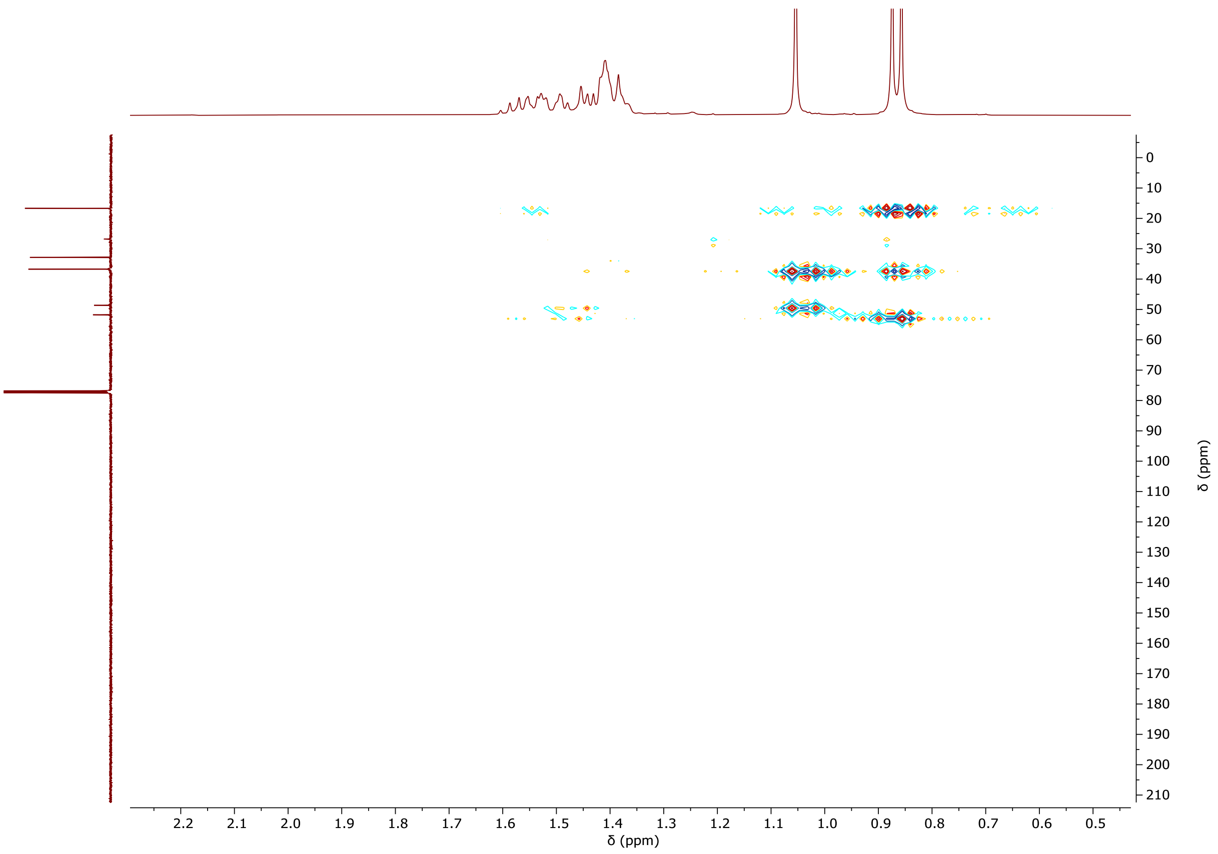

Figure S26. $^{1}H^{13}C$ HMBC NMR spectrum of **3** in $CDCl_3$.

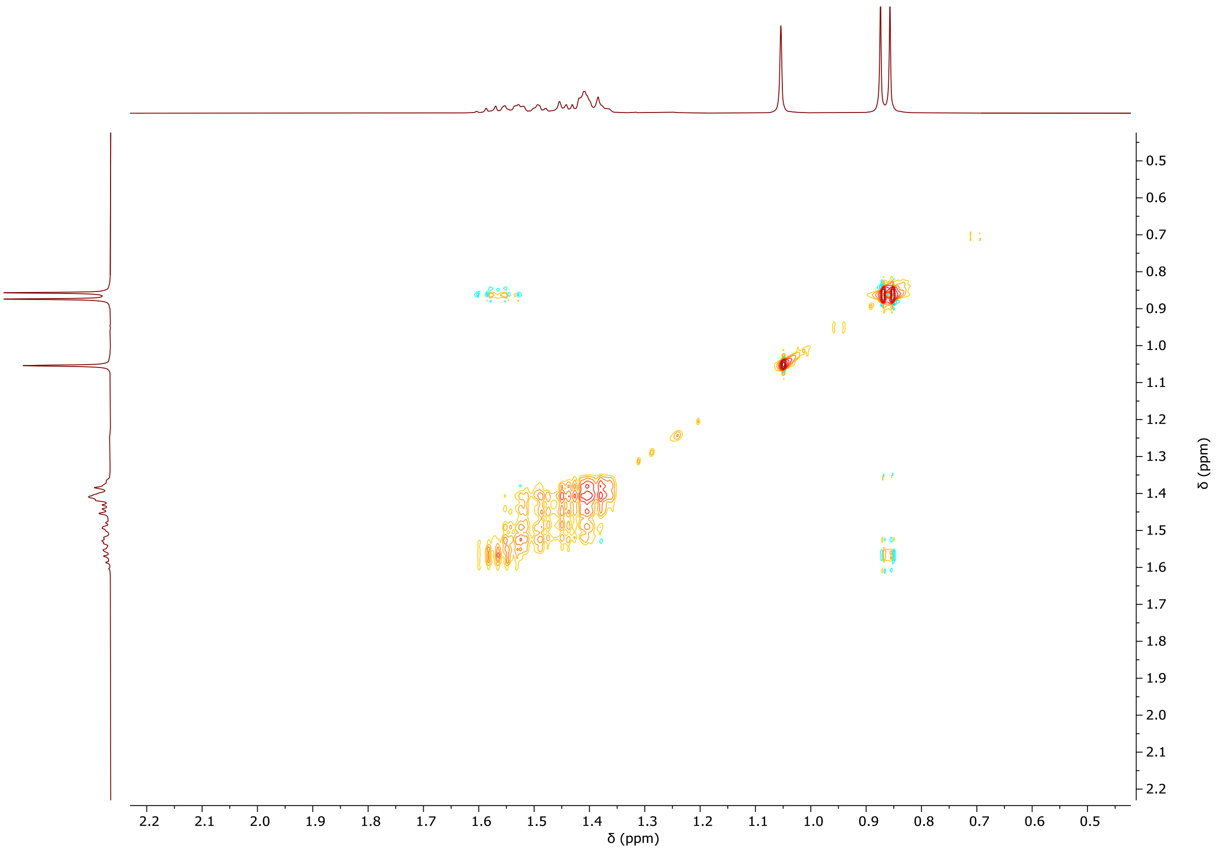

Figure S27. $^{1}H^{1}H$ NOESY NMR spectrum of **3** in $CDCl_3$.

4.2 IR spectra

Dibenzyl (1R,4R)-1-isopropyl-4-methyl-2,3-diazabicyclo[2.2.2]oct-5-ene-2,3-dicarboxylate (**2**)

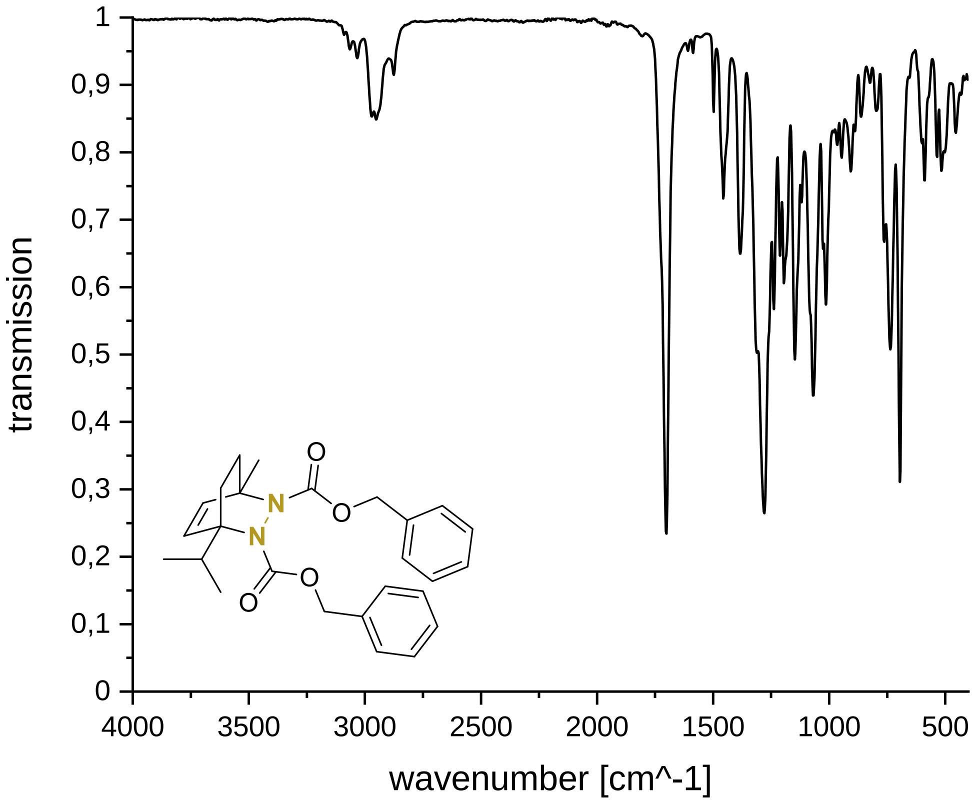

Figure S28. FT-IR spectrum of **2a**.

Dibenzyl 1-(5-isopropyl-2-methylcyclohexa-2,5-dien-1-yl)hydrazine-1,2-dicarboxylate (**2b**):

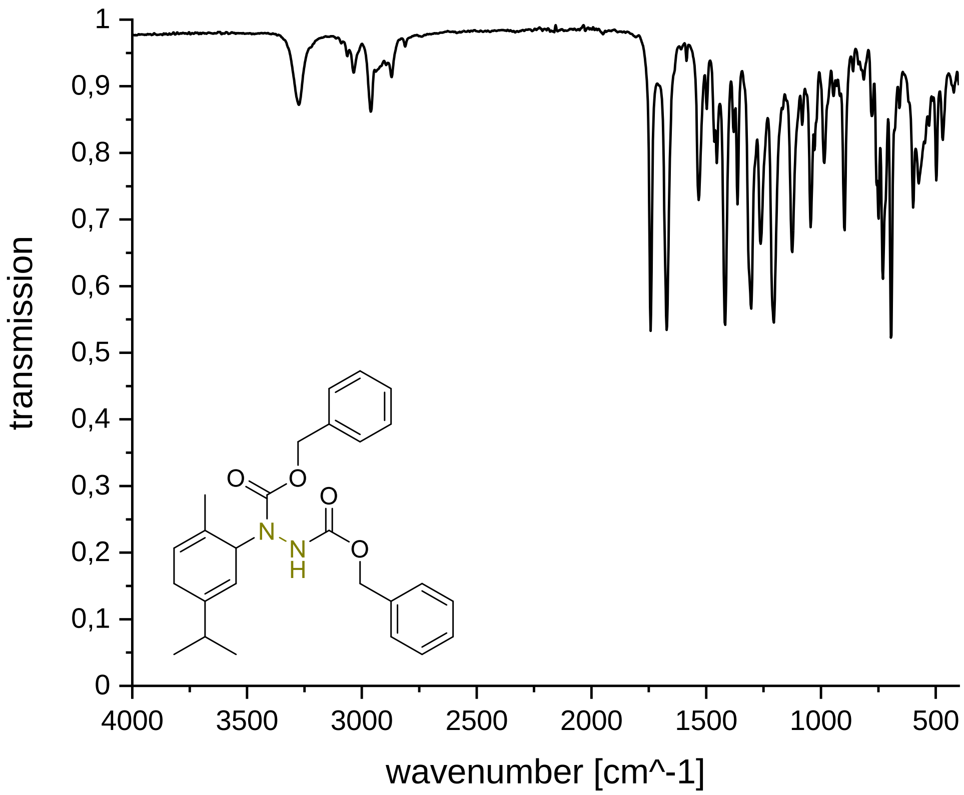

Figure S29. FT-IR spectrum of **2b**.

1,4-*p*-Menthane diamine (**3**)

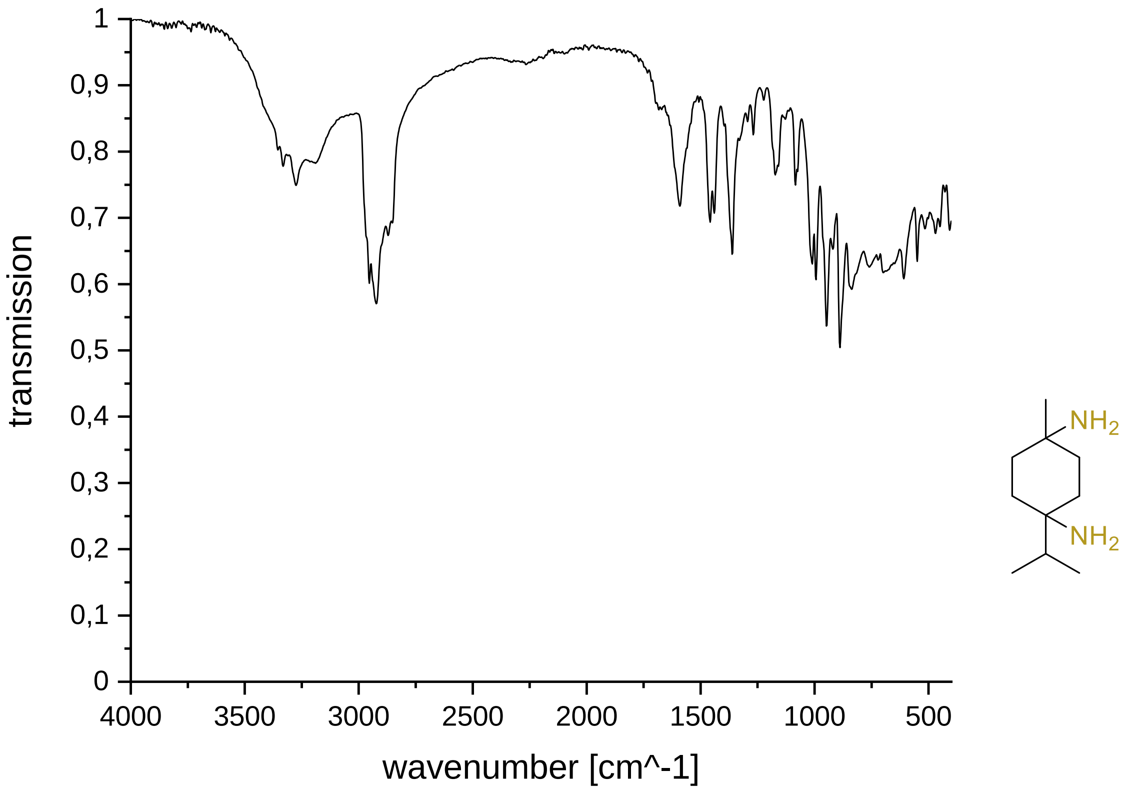

Figure S30. FT-IR spectrum of **3**.

5 Crystallographic data

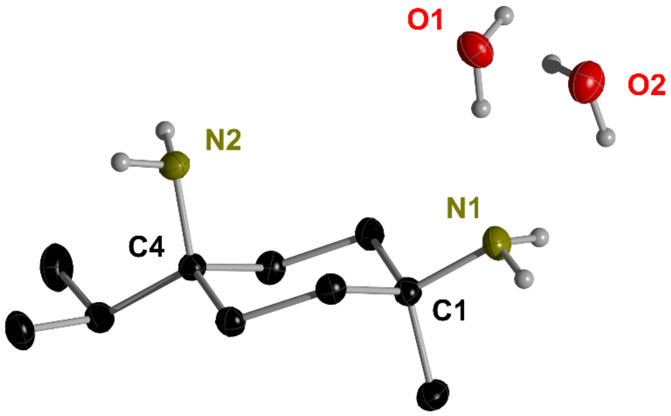

Figure S31. SC-XRD structure of 3 • 2 H_2O. Thermal ellipsoids at 30% probability level. Carbon-bonded hydrogen atoms are omitted for clarity.

5 Crystallographic data

Table S4. Crystal data and structure refinement for 1,4-*p*-menthane diamine (**3**).

**compound**	1,4-*p*-menthane diamine (**3**)
CCDC #	2325396
Empirical formula	$C_{10}H_{26}N_2O_2$
Formula weight	206.33
Temperature/K	200
Crystal system	monoclinic
Space group	C2/c
a / Å	25.4213(14)
b / Å	5.9035(2)
c / Å	16.5324(9)
α / °	90
β / °	90.618(4)
γ / °	90
Volume/Å^3	2481.0(2)
Z	8
ρ_{calc}g/cm^3	1.105
μ / mm^{-1}	0.076
F(000)	928.0
Crystal size/mm^3	0.4 × 0.367 × 0.3
Radiation	Mo Kα (λ = 0.71073)
Θ_{min} – Θ_{max} / °	3.204 to 56.99
Reflections collected	9535
Independent reflections	3146
R_{int}	0.0199
Data/restraints/parameters	3146/6/155
GooF	1.064
R_1	0.0353
wR_2	0.0996
R_1(all)	0.0457
wR_2(all)	0.1035
ρ_{e-} max/ρ_{e-} min/ e Å^{-3}	0.23/-0.17

6 References

[1] P.-K. Dannecker, M. A. R. Meier, *Sci. Rep.* **2019**, *9*, 9858.
[2] W. C. Still, M. Kahn, A. Mitra, *J. Org. Chem.* **1978**, *43*, 2923.
[3] G. R. Fulmer, A. J. M. Miller, N. H. Sherden, H. E. Gottlieb, A. Nudelman, B. M. Stoltz, J. E. Bercaw, K. I. Goldberg, *Organometallics* **2010**, *29*, 2176.
[4] J. Koziskova, F. Hahn, J. Richter, J. Kožíšek, *Acta Chim. Slov.* **2016**, *9*, 136.
[5] O. V. Dolomanov, L. J. Bourhis, R. J. Gildea, J. A. K. Howard, H. Puschmann, *J. Appl. Crystallogr.* **2009**, *42*, 339.
[6] G. M. Sheldrick, *Acta Crystallogr. A Found. Adv.* **2015**, *71*, 3.
[7] G. M. Sheldrick, *Acta Crystallogr. C Struct. Chem.* **2015**, *71*, 3.
[8] US 10519100B1, **2018**.
[9] M. Firdaus, M. A. R. Meier, *Green Chem.* **2013**, *15*, 370.
[10] A. Causero, C. Troll, B. Rieger, *Ind. Eng. Chem. Res.* **2020**, *59*, 15464.
[11] a) S. S. Kovals'skaya, N. G. Kozlov, T. S. Tikhonova, *Chem. Nat. Compd.* **1989**, *25*, 552; b) S. Zhu, S. Xu, X. Yi, J. Wang, Z. Zhao, J. Jiang, *Ind. Crops Prod.* **2018**, *115*, 111; c) Jing Wang, Shichao Xu, Xiaojing Zeng, Huanhuan Dong, Jianxin Jiang, Zhendong Zhao, *Linchan Huaxue Yu Gongye/Chem* **2018**, *38*, 59; d) CN 102040525 A, **2013**; e) CN 113200872 A, **2021**; f) CN 101085740 A, **2009**; g) CN 104529790 A, **2015**; h) Zhi-yong Peng, Dong-mei Li, Zhen-dong Zhao, Liang-wu Bi, Jing Wang, Yan Gu, *Linchan Huaxue Yu Gongye/Chem* **2008**, *28*, 16; i) US2955138 A, **1960**.
[12] D. Jaramillo, D. P. Buck, J. G. Collins, R. R. Fenton, F. H. Stootman, N. J. Wheate, J. R. Aldrich-Wright, *Eur. J. Inorg. Chem.* **2006**, *2006*, 839.
[13] a) *Org. Synth.* **2002**, *79*, 125; b) F. R. Kinzl, H. M. Riepl, *Helv. Chim. Acta* **2015**, *98*, 447; c) J. Ackrell, M. Altaf-ur-Rahman, A. J. Boulton, R. C. Brown, *J. Chem. Soc., Perkin Trans. 1* **1972**, 1587.
[14] DE3135948 A1, **1983**.
[15] H. Blattmann, R. Mülhaupt, *Green Chem.* **2016**, *18*, 2406.
